# White matter hyperintensities are associated with disproportionate progressive hippocampal atrophy

**DOI:** 10.1002/hipo.22690

**Published:** 2017-01-09

**Authors:** Cassidy M. Fiford, Emily N. Manning, Jonathan W. Bartlett, David M. Cash, Ian B. Malone, Gerard R. Ridgway, Manja Lehmann, Kelvin K. Leung, Carole H. Sudre, Sebastien Ourselin, Geert Jan Biessels, Owen T. Carmichael, Nick C. Fox, M. Jorge Cardoso, Josephine Barnes, for the Alzheimer's Disease Neuroimaging Initiative

**Affiliations:** ^1^ Department of Neurodegenerative DiseaseDementia Research Centre, UCL Institute of NeurologyLondonUnited Kingdom; ^2^ Statistical Innovation GroupCambridgeAstraZenecaUnited Kingdom; ^3^ Translational Imaging GroupCentre for Medical Image Computing, University College LondonLondonUnited Kingdom; ^4^ Nuffield Department of Clinical NeurosciencesFMRIB Centre, University of OxfordUnited Kingdom; ^5^ Wellcome Trust Centre for NeuroimagingLondonUnited Kingdom; ^6^ Department of Neurology and NeurosurgeryBrain Center Rudolf Magnus University Medical Center UtrechtThe Netherlands; ^7^ Pennington Biomedical Research CenterBaton RougeLouisiana

**Keywords:** Alzheimer's disease, vascular disease, mild cognitive impairment, hippocampus, white matter hyperintensity (WMH), white matter disease

## Abstract

This study investigates relationships between white matter hyperintensity (WMH) volume, cerebrospinal fluid (CSF) Alzheimer's disease (AD) pathology markers, and brain and hippocampal volume loss. Subjects included 198 controls, 345 mild cognitive impairment (MCI), and 154 AD subjects with serial volumetric 1.5‐T MRI. CSF Aβ_42_ and total tau were measured (*n =* 353). Brain and hippocampal loss were quantified from serial MRI using the boundary shift integral (BSI). Multiple linear regression models assessed the relationships between WMHs and hippocampal and brain atrophy rates. Models were refitted adjusting for (a) concurrent brain/hippocampal atrophy rates and (b) CSF Aβ_42_ and tau in subjects with CSF data. WMH burden was positively associated with hippocampal atrophy rate in controls (*P =* 0.002) and MCI subjects (*P =* 0.03), and with brain atrophy rate in controls (*P =* 0.03). The associations with hippocampal atrophy rate remained following adjustment for concurrent brain atrophy rate in controls and MCIs, and for CSF biomarkers in controls (*P* = 0.007). These novel results suggest that vascular damage alongside AD pathology is associated with disproportionately greater hippocampal atrophy in nondemented older adults. © 2016 The Authors Hippocampus Published by Wiley Periodicals, Inc.

## INTRODUCTION

Atrophy rate, or rate of tissue loss over time, is one of the markers that reflects disease progression and severity in Alzheimer's disease (AD) (Fox et al., 1999; Mungas et al., 2002; Jack et al., 2005). Hippocampal atrophy is an early marker of AD pathology (Henneman et al., 2009; Dubois et al., 2014); rates correlate with cerebrospinal fluid (CSF) markers of AD (Hampel et al., 2005; Schuff et al., 2009) and also with cognitive decline (Jack et al., 2000). In clinical trials with cognitive outcomes, it has been suggested that sample size can be reduced by enriching the sample with MCI subjects displaying an atrophy pattern involving the hippocampus (McEvoy et al., 2010; Yu et al., 2014). However, atrophic hippocampi and elevated hippocampal atrophy rates could have multiple causes and may not be solely attributable to underlying AD pathology.

Cerebral vascular pathology is an important cause of cognitive decline, and although criteria exist to support a diagnosis of AD or vascular dementia (Mckhann et al., 1984; Román et al., 1993), differentiation of these causes is difficult and mixed pathology is often present (Schneider et al., 2007). Many AD cases have vascular damage that is visible on brain imaging (Yoshita et al., 2006) and apparent at autopsy (Fernando and Ince, 2004; Jellinger and Attems, 2007). One type of vascular damage appears hypointense on T1‐weighted MRI and hyperintense on T2‐weighted imaging; so‐called white matter hyperintensities (WMHs) can be quantified volumetrically (Prins et al., 2004; Carmichael et al., 2010; Schmidt et al., 2012). WMH volume is associated with increasing age (Yoshita et al., 2006), risk of future cognitive decline and dementia (van der Flier et al., 2005; Carmichael et al., 2010). WMHs are associated with numerous potentially modifiable cerebrovascular risk factors (Debette et al., 2011).

Whilst WMHs have been found to associate with longitudinal brain volume changes (Enzinger et al., 2005; Schmidt et al., 2005; Barnes et al., 2013), their relevance to medial temporal lobe (MTL) atrophy is less clear; some have reported positive correlations between WMH and MTL atrophy (Eckerström et al., 2011; Ye et al., 2014; Crane et al., 2015; Knopman et al., 2015) and others have not (Du et al., 2006; Rossi et al., 2006; Van De Pol et al., 2007; Ota et al., 2011; Gattringer et al., 2012; Raji et al., 2012; Nosheny et al., 2015). Such varying findings may be due to key differences in the level of cognitive impairment and vascular risk burden of subjects investigated. Few studies have investigated across the disease spectrum, and no study has thoroughly investigated whether the relationship between WMHs and hippocampal atrophy rate is explained by the relationship of AD pathology; which is likely to be important in cognitively impaired subjects. Improved understanding of the factors that may underlie hippocampal atrophy rates is crucial for the design of effective clinical trials aimed at preventing AD where atrophy rates are used as outcome measures. As trials are being implemented at earlier stages of disease, i.e. in prodromal/presymptomatic individuals, it is important to understand these interrelationships at the earliest stages.

Following on from a previous study investigating longitudinal whole‐brain atrophy rate and WMHs (Barnes et al., 2013), we investigated the relationship between WMHs and atrophy of the hippocampus. In order to disentangle the associations of WMHs and hippocampal atrophy from AD pathology, we also adjusted our analyses for CSF biomarkers and concurrent brain atrophy. Our primary measure of atrophy was estimated using the boundary shift integral (BSI); to see if observed relationships between WMHs and atrophy were substantiated using a second technique we implemented cross‐sectional and longitudinal voxel‐based morphometry (VBM). Our specific hypotheses were:

1. WMH volume is related to hippocampal atrophy; greater WMH is associated with smaller cross‐sectional hippocampal volume and greater longitudinal reduction in hippocampal volume in control, MCI, and AD subjects.
2. Among brain regions, the hippocampus is differentially vulnerable to vascular damage; the relationship between WMH volume and hippocampal volume change will remain following correction for concurrent whole‐brain volume change.
3. The hippocampal atrophy is related to vascular damage; the relationship between WMHs and hippocampal volume change will remain following adjustment for CSF biomarkers of tau and amyloid beta 1‐42 (Aβ_42_).

VBM analyses were also used to explore whether WMHs were associated with tissue loss in any extra‐hippocampal areas of the brain.

## MATERIALS AND METHODS

### Subjects

All data used in this study were obtained from the Alzheimer's Disease Neuroimaging Initiative (ADNI1) database (http://www.loni.usc.edu/). Launched in 2003, ADNI is a multicentre, private/public funded longitudinal study investigating healthy adults, MCI and AD subjects and is led by Principle Investigator Michael W. Weiner, MD. Its primary goal is to test whether serial magnetic resonance imaging (MRI), positron emission tomography (PET), other biological markers, and clinical and neuropsychological assessment can be combined to measure AD progression. For up‐to‐date information, see www.adni-info.org.

**Figure 1:**
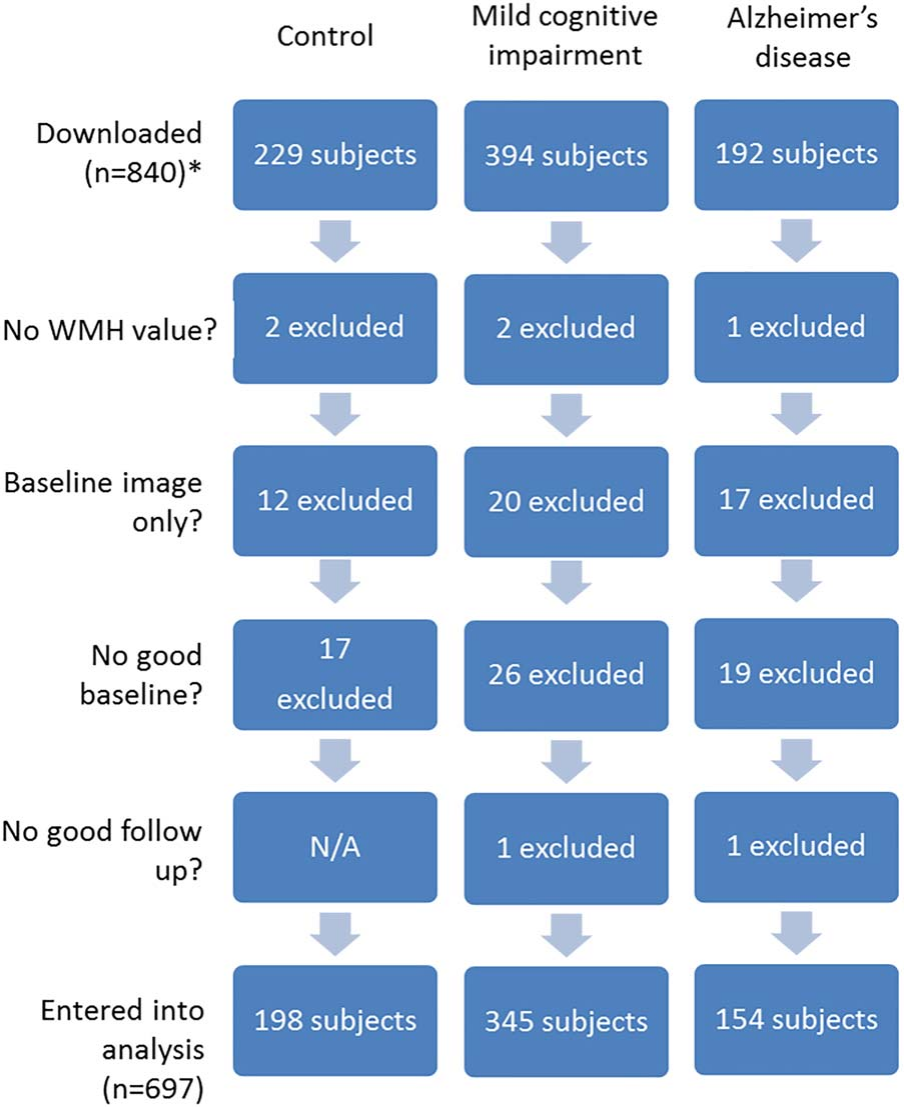
Flowchart showing the subject selection process for the cross‐sectional and longitudinal analysis, by diagnostic group. *Twenty‐five subjects were additionally excluded which had no available diagnosis; scans were failed at initial scan by LONI. [Color figure can be viewed at wileyonlinelibrary.com]

Written informed consent was obtained as approved by the Institutional Review Board at each participating centre. Participants took part in baseline clinical, neuropsychometric and MRI assessments, and periodical assessments thereafter, the frequency of which varied dependent on the diagnostic group. CSF data were collected in a proportion of subjects, details of the Aβ_42_ and tau analysis have been described previously (Shaw et al., 2009). We analysed data from control, MCI and AD subjects from ADNI1 who had a baseline 1.5‐T MRI scan and at least 1 follow‐up 1.5‐T MRI scan. Following quality control 143 scans were excluded (see Figure 1); of which 22% had a diagnosis of control, 34% of MCI, 27% of AD, and 17% had no diagnostic information available (these scans were failed at initial visit by LONI).

### Image Acquisition and Assessment

The ADNI MRI protocol is described in detail elsewhere (Jack et al., 2008). Following acquisition, each image underwent quality control at the Mayo Clinic (Rochester, MN) which included protocol compliance check, inspection for clinically significant medical abnormalities, and image quality assessment. Pre‐processing steps using the standard ADNI image processing pipeline were then applied, including gradient warping (Jovicich et al., 2006), B1 nonuniformity (Narayana et al., 1988) and intensity nonuniformity correction (Sled et al., 1998). These preprocessed images underwent internal quality control at the Dementia Research Centre, London, UK. Images with significant motion artefacts causing severe blurring at the tissue boundaries, were excluded from this study.

### Hippocampal and Whole‐brain Volumes and Rates of Change

Imaging data consisted of 0‐, 6‐, 12‐, 18‐, 24‐, and 36‐month scans where available. Brain volumes were estimated from the 1.5‐T volumetric T1‐weighted images using a multi atlas template brain segmentation method (Leung et al., 2011). Hippocampal volumes were estimated automatically using a validated multi atlas template method (Leung et al., 2010). The left and right hippocampal values were summed together. The symmetric boundary shift integral (BSI), was used to estimate change directly from scan pairs following segmentation (Leung et al., 2012), the outcome representing ml of brain or hippocampus lost during the scan interval. All registrations were visually checked and excluded in cases of severe motion or warp due to positional differences. Total Intracranial Volumes (TIV) were calculated using the SPM 12 package (Statistical Parametric Mapping; http://www.fil.ion.ucl.ac.uk/spm) by summing the grey matter (GM), white matter (WM) and cerebrospinal fluid (CSF) tissue segmentations (Malone et al., 2015). The technique used was previously found to correlate well with manual segmentation in AD patients (*R* ^2^ = 0.940, 95% confidence interval, 95% CI = 0.924–0.953) (Malone et al., 2015). Voxels representing WMH were included in TIV estimation. WMH volumes were segmented from baseline PD, T1, and T2 images using an automated technique, checked by a trained analyst and edited for gross errors (Schwarz et al., 2009; Carmichael et al., 2010); these values were downloaded from the ADNI website (http://www.loni.usc.edu/). WMH volumes were log transformed (base 2) to reduce skewness, its estimated coefficient thus represents the expected change in atrophy rate corresponding to a doubling of WMH volume on the original scale.

### Statistical Methods

#### Group demographics

To test for differences in baseline variables between diagnostic groups linear regression (ANOVA) was used for age, MMSE, total brain volume, total hippocampal volume, TIV and log_2_WMH. TIV was used as a covariate when estimating differences in baseline hippocampal, brain and log_2_WMH volume. To look for differences between groups in gender, hypertension, hypercholesterolemia, diabetes, smoking, and presence of an APOE e4 allele, Fisher's exact test was used.

#### Cross‐sectional hippocampal and whole brain volumes

All cross‐sectional and longitudinal analyses were performed for the whole‐brain and hippocampus, separately in each diagnostic group.

To analyse the cross‐sectional relationship between volumes and baseline WMH volume a multiple linear regression was performed with baseline volume (hippocampal or whole‐brain) as the outcome, log_2_WMH as the predictor variable, and TIV and gender as covariates.

#### Longitudinal hippocampal and whole volume changes

To analyse the association between baseline log_2_WMH volume and whole‐brain and hippocampal atrophy rates, linear mixed effects models were fitted. The dependent variable was the BSI (ml of brain or hippocampus lost during the scan interval). Interval (years) between baseline and follow‐up scans was included as a fixed effect such that the resulting coefficient estimated from the model represents the change of volume in ml per year. Interval was also included as a random effect to permit between subject heterogeneity in atrophy rate; the trajectories of atrophy rate for each individual were able to vary, to allow for unknown factors which influence atrophy rate between individuals. An interaction between baseline log_2_WMH volume and interval allowed for the former to influence mean atrophy rate. No intercept was included in the model; ensuring that the estimated atrophy rate over a scan interval of zero is zero.

In order to estimate the association between baseline log_2_ WMH and hippocampal volume change whilst adjusting for whole‐brain atrophy (and vice versa), a joint mixed effects model was constructed, see Manning et al., 2014. This model permits the random effects dictating the rates of hippocampal and whole‐brain atrophy rates to be correlated. It accommodates missing values under the missing at random assumption (Manning et al., 2014). Models were additionally run allowing for CSF biomarkers to modify atrophy rates. In additional analyses age and vascular risk factors (VRFs) were added to the longitudinal models. These covariates were added as interaction terms with interval, to investigate whether their addition affected relationships between WMH and hippocampal atrophy rate. The VRFs added were history of hypertension, hypercholesterolemia, diabetes, smoking, and APOE genotype (presence of one or more APOE e4 alleles). As the dependent variable represents absolute volume change, all analyses were adjusted for TIV, also using an interaction with interval. We also investigated whether the effect of WMH on atrophy rates was dependent on TIV to test whether subjects with the same volume of WMH, but different TIVs (serving as a proxy for head size) have differing relationships between WMH and atrophy. For this an interaction term between TIV, WMH, and interval was added to the previous mixed models of WMH and TIV with atrophy rate as outcome. Consequently both atrophy rate and WMH were adjusted for TIV; the former through the main effect of TIV and the latter with the WMH‐TIV interaction term. The overall effect of WMH on atrophy rates with the TIV‐WMH interaction was tested by comparing models with the WMH‐TIV interaction and WMH as predictors, versus models without these coefficients using a likelihood ratio test.

As most subjects had multiple BSI values from their longitudinal visits, we generated a single atrophy rate estimate per person from a mixed effects regression model without adjustment for covariates except scan interval; these were used to generate longitudinal plots. Postestimation linear predictions (mean rate plus predicted individual random effect) of rate were plotted against log_2_WMH for visualization purposes. Simple regression lines were overlaid on the plots.

#### Voxel‐based morphometry (VBM)

Cross‐sectional and longitudinal VBM was used to assess the correlation between log_2_WMH and patterns of volume change in GM and WM. Subjects with a usable baseline and 24‐month scan pair were used, the VBM dataset thus represents a proportion of the original BSI dataset (456 subjects of 697 used for BSI). For cross‐sectional analysis, baseline images were first rigidly reorientated to Montreal Neurological Institute (MNI) space, segmented into GM, WM and CSF, and normalised to a groupwise space (DARTEL). Segments were smoothed with a 6mm Gaussian kernel and a groupwise mask was created for analysis (Ridgway et al., 2009). See Supporting Information Methods for more information.

For longitudinal analysis, baseline and 24‐month images were first independently rigidly reoriented to MNI space. Using SPM12's Pairwise Longitudinal Registration tool baseline and 24‐month images were registered, producing a midpoint average image (Ashburner and Ridgway, 2013). Voxel‐wise volume change maps were quantified as the difference between follow‐up and baseline Jacobian maps (which measure the volume change relative to, and in the space of, the average image) divided by the scan interval. Segmentation and DARTEL normalization as above were performed using the midpoint average image. Volume change maps were smoothed using Tissue Weighted Smoothing, separately for GM and WM. All tests were thresholded to correct for multiple comparisons, controlling the Family Wise Error (FWE) at a level of *p* < 0.05. See Supporting Information Methods for more information.

## RESULTS

### Group Demographics

Data from 840 subjects were downloaded from the ADNI website. Subjects were excluded if they had a baseline scan only, were missing WMH values and/or failed internal quality control (see Image Acquisition and Assessment section), see Figure 1. Table 1 shows demographic and image summary statistics from the 697 subjects included in the analysis. There were statistically significant differences between the three diagnostic groups in gender distribution, with a greater proportion of male subjects in the MCI group. Subject groups also differed in MMSE, total brain volume, total hippocampal volume, APOE genotype and WMH volume at baseline in a manner consistent with MCI and AD. See Supporting Information Results Table 1 for demographic differences in the VBM subset.

**Table 1:** Subject Demographics and Basic Imaging Information

	Controls	MCI	AD	*p* value across groups
*N* (total = 697)	198	345	154	
Age at baseline (yr)	75.9 (5.1)	75.0 (7.2)	75.0 (7.7)	0.3
Percentage male	52.5	63.2	53.9	0.03
MMSE at baseline, /30	29.1 (1.0)	27.0 (1.8)	23.4 (1.9)	<0.001
Length of follow‐up (yr); minimum, maximum	2.6 (0.8); 0.5, 3.7	2.3 (0.8); 0.5, 3.5	1.7 (0.6); 0.5, 3.1	<0.001
BSI measurements per subject, No.; minimum, maximum	3.2 (0.9); 1, 4	3.6 (1.3); 1, 5	2.3 (0.8); 1, 3	<0.001
Total brain volume (ml)	1,068 (103)	1,061 (115)	1,022 (115)	<0.001^a^
Total hippocampal volume (ml)	5.2 (0.7)	4.5 (0.8)	3.9 (0.9)	<0.001^a^
Total intracranial volume (ml)	1,445 (134)	1,468 (146)	1,450 (163)	0.2
White matter hyperintensity (ml); log_2_WMH (ml)	0.24 (0.5); −2.37 (2.3)	0.28 (0.6); −2.08 (2.4)	0.40 (1.0); −1.37 (2.2)	<0.001^a^
Percentage hypertensive	42	50	52	0.1
Percentage hypercholesteraemic	25	30	36	0.1
Percentage diabetic	6	7	6	0.8
Percentage APOE e4 carrier	26	55	69	<0.001
Percentage smoker (past or current)	40	41	40	1.0

Values are mean (SD) unless reported. White matter hyperintensity values reported as median with IQR.

a Adjusted for TIV. APOE e4 carrier refers to individuals with one or more APOE e4 alleles.

### Cross‐Sectional Analyses

Table 2 shows the partial regression coefficients for the association between WMHs and cross‐sectional brain and hippocampal baseline volumes. Average brain volumes calculated from the model were 1,071 ml in control subjects, 1,068 ml in MCI subjects and 1,024 ml in AD subjects for females with mean TIV and WMH values. Average hippocampal volumes were 5.2 ml for controls, 4.4 for MCI subjects, and 3.9 ml for AD subjects for females with mean TIV and WMH values. There was strong evidence of an association between WMH burden and baseline brain volume in MCI and AD groups, after adjusting for gender and TIV. Each doubling of WMH burden was estimated to be associated with a smaller brain volume of 6.0 ml (95% CI 3.4–8.5), and 6.6 ml (2.4–10.9) in the MCI and AD groups, respectively. In controls there was borderline statistically significant evidence of an association, with a doubling of WMH associated with a brain volume of 3.4 ml (0.1–6.8) less than average brain volume. There was strong evidence of an association between WMHs and baseline hippocampal volume in all three groups, with a doubling of WMH volume associated with a 0.06 (0.02–0.10), 0.08 (0.05–0.12), and 0.09 ml (0.03–0.15) reduction in the average control, MCI and AD hippocampal volumes respectively. There were no significant relationships between gender and either whole‐brain or hippocampal volume.

**Table 2:** Results From the Regression Models Assessing the Relationship Between Cross‐Sectional Brain and Hippocampal Volumes (Outcome Measures) and log2WMH Volume (Predictor)

	Controls	MCI	AD
*N*	198	345	154
Mean brain volume adjusted for WMH, TIV and gender (ml)	1,071.1	1,067.6	1,024.0
Mean hippocampal volume adjusted for WMH, TIV and gender (ml)	5.2	4.4	3.9
Association between WMH and baseline brain volume	−3.41 (−6.76 to −0.06); *p =* 0.05	−5.95 (−8.48 to −3.42); *p <* 0.001	−6.61 (−10.87 to −2.35); *p =* 0.003
Association between WMH and baseline hippocampal brain volume	−0.06 (−0.10 to −0.02); *p =* 0.003	−0.08 (−0.12 to −0.05); *p <* 0.001	−0.09 (−0.15 to −0.03); *p =* 0.003

Estimates are shown with 95% confidence intervals for an increase in volume (ml) for a doubling of WMH, conditional on intracranial volume and gender.

### Longitudinal Analyses

**Figure 2:**
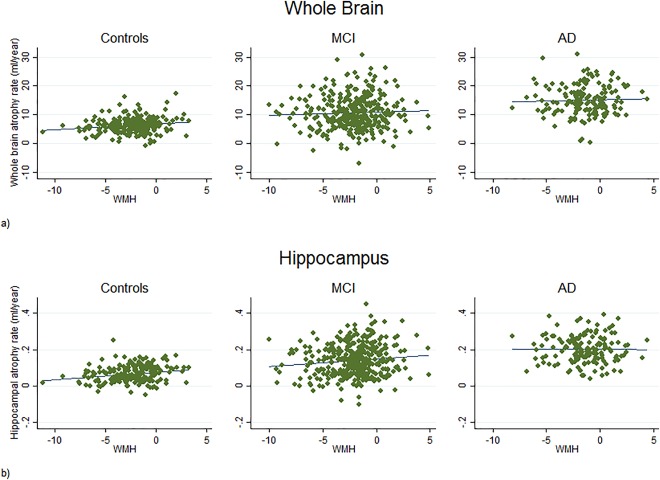
Plots showing the relationship between baseline log2WMH, whole‐brain (a), and hippocampal atrophy rate (b) in control, MCI and AD subjects. Individual predicted atrophy rate estimated from mixed model with predictors of BSI (tissue loss at all scanning intervals) and scan interval. [Color figure can be viewed at wileyonlinelibrary.com]

Table 3 shows the partial regression coefficients for the association between baseline WMHs, brain atrophy rates and hippocampal atrophy rates. Figure 2 shows scatterplots of whole‐brain and hippocampal atrophy rates against log_2_WMH. Mean atrophy rates of the whole‐brain were estimated to be 6.3 (5.8–6.8), 10.7 (10.0–11.4), 15.1 (14.0–16.1) ml/year for control, MCI and AD subjects respectively for subjects with mean TIV values. Average hippocampal atrophy rates were estimated to be 0.07 (0.06–0.07) ml/year in the control group, 0.1 (0.13–0.15) ml/year in the MCI group, and 0.2 (0.19–0.22) ml/year in AD subjects with mean TIV values. There was some evidence of an association between baseline WMHs and subsequent whole‐brain atrophy rate in controls, with each doubling in WMH volume associated with an estimated increase in atrophy rate of 0.3 ml/year (0.03–0.5). There was stronger evidence for an association with hippocampal atrophy in controls, with each doubling of WMH volume estimated to increase atrophy by 0.005 ml/year (0.002–0.009). The association between WMHs and whole‐brain atrophy in controls was substantially reduced after adjusting for concurrent hippocampal atrophy, with the independent effect reduced to 0.06 ml/year (from 0.3 ml/year), and was no longer statistically significant after adjustment. The association between WMHs and hippocampal atrophy was also materially reduced in controls after adjusting for concurrent whole‐brain atrophy, although there remained statistically significant evidence of an independent association, with each doubling of WMH volume associated with a 0.003 ml/year (0.0003–0.006) increase in hippocampal atrophy rate. In patients with MCI, the association between WMHs and hippocampal atrophy was also reduced after adjustment for concurrent whole‐brain atrophy, with each doubling of WMHs associated with a 0.003 ml/year (0.0004–0.007) increase in hippocampal rate. Other associations were not statistically significant.

**Table 3:** Results From the Regression Models Assessing the Relationship Between Atrophy Rates (Outcome Measures) and log2WMH Volume (Predictor)

	Controls	MCI	AD
*N*	198	345	154
Rate of whole‐brain atrophy (ml/yr)	6.31 (5.82 to 6.79) *p <* 0.001	10.69 (10.00 to 11.37) *p <* 0.001	15.08 (14.02 to 16.14) *p <* 0.001
Rate of hippocampal atrophy (ml/yr)	0.07 (0.06 to 0.07); *p <* 0.001	0.14 (0.13 to 0.15); *p <* 0.001	0.20 (0.19 to 0.22); *p <* 0.001
Association between WMH and whole‐brain atrophy^a^	0.3 (0.03 to 0.5); *p =* 0.03	0.1 (−0.2 to 0.4); *P =* 0.4	0.06 (−0.4 to 0.5); *p =* 0.8
Association between WMH and hippocampal atrophy^a^	0.005 (0.002 to 0.009); *p =* 0.002	0.005 (0.0004 to 0.009); *p =* 0.03	−0.0005 (−0.008 to 0.007); *p =* 0.9
Association between WMH and whole‐brain atrophy rate adjusted for hippocampal atrophy rate^b^	0.06 (−0.1 to 0.3); *p =* 0.5	−0.1 (−0.3 to 0.1); *p =* 0.3	0.07 (−0.3 to 0.5); *p =* 0.7
Association between WMH and hippocampal atrophy rate adjusted for whole‐brain atrophy rate^c^	0.003 (0.0003 to 0.006); *p =* 0.03	0.003 (0.0004 to 0.007); *p =* 0.03	−0.0009 (−0.007 to 0.005); *p =* 0.8

a Estimates are shown for increase in atrophy rate (ml/year), with 95% confidence intervals: for a doubling of WMH, conditional on intracranial volume.

b Estimates are shown for increase in atrophy rate (ml/year), with 95% confidence intervals: for a, conditional on intracranial volume and hippocampal atrophy rate

c Estimates are shown for increase in atrophy rate (ml/year), with 95% confidence intervals: for a conditional on intracranial volume and whole‐brain atrophy rate.

### CSF Subset Analyses

#### Associations between log_2_WMH and atrophy rates following CSF biomarker adjustment

Table 4 shows the results from the biomarker analyses. The relationship between whole‐brain and hippocampal atrophy and log_2_WMH remained statistically significant in the control group following adjustment for CSF biomarkers, with a doubling of WMH volume associated with an increase in atrophy rate of 0.3 ml/year (0.07–0.6) for the whole‐brain and 0.008 ml/year (0.004–0.01) for the hippocampus. There was no evidence of an association between WMHs and hippocampal atrophy rate after CSF biomarker adjustment in MCI subjects. Importantly, in the control group a strong association between WMHs and hippocampal atrophy rate survived following adjustment for concurrent whole‐brain atrophy rate and CSF biomarkers, with a doubling in WMH volume estimated to increase hippocampal atrophy rate by 0.005 ml/year (0.001–0.009). However, it did not remain statistically significant in the subset of MCI patients with CSF measures, in contrast to findings in the whole group unadjusted for CSF biomarkers.

**Table 4:** Results From the Regression Models Assessing the Relationship Between Atrophy Rates (Outcome Measures) and log2WMH Volume, CSF Aβ, and tau (Predictors)

	Controls	MCI	AD
*N*	100	167	86
Rate of whole‐brain atrophy (ml/yr)	6.25 (5.64 to 6.86); *p <* 0.001	11.46 (10.53 to 12.40); *p <* 0.001	14.48 (13.18 to 15.79); *p <* 0.001
Rate of hippocampal atrophy (ml/yr)	0.06 (0.05 to 0.07); *p <* 0.001	0.15 (0.14 to 0.16); *p <* 0.001	0.19 (0.17 to 0.21); *p <* 0.001
Association between WMH and whole‐brain atrophy rate^a^	0.3 (0.07 to 0.6); *p =* 0.01	0.2 (−0.2 to 0.6); *p =* 0.4	−0.2 (−0.8 to 0.3); *p =* 0.4
Association between WMH and hippocampal atrophy rate^a^	0.008 (0.004 to 0.01); *p <* 0.001	0.004 (−0.002 to 0.01); *p =* 0.2	−0.0008 (−0.008 to 0.007); *p =* 0.8
Association between CSF Aβ_42_ and whole‐brain atrophy rate^b^	−0.2 (−0.3 to −0.06); *p =* 0.004	−0.4 (−0.6 to −0.2); *p <* 0.001	−0.3 (−0.6 to 0.03); *p =* 0.08
Association between CSF Aβ_42_ and hippocampal atrophy rate^b^	−0.001 (−0.003 to 0.0008); *p =* 0.2	−0.006 (−0.009 to −0.003); *p <* 0.001	−0.004 (−0.008 to 0.0003); *p =* 0.07
Association between CSF tau and whole‐brain atrophy rate^c^	0.2 (−0.04 to 0.4); *p =* 0.1	0.04 (−0.2 to 0.2); *p =* 0.7	0.1 (−0.1 to 0.3); *p =* 0.4
Association between CSF tau and hippocampal atrophy rate^c^	0.005 (0.002 to 0.009); *p =* 0.006	0.002 (−0.001 to 0.005); *p =* 0.3	−0.001 (−0.004 to 0.002); *p =* 0.6
Association between WMH and whole‐brain atrophy rate adjusted for hippocampal atrophy rate^d^	0.1 (−0.1 to 0.3); *p =* 0.4	−0.004 (−0.3 to 0.3); *p* > 0.9	−0.2 (−0.7 to 0.3); *p =* 0.5
Association between WMH and hippocampal atrophy rate adjusted for whole‐brain atrophy rate^e^	0.005 (0.001 to 0.009); *p =* 0.007	0.002 (−0.002 to 0.007); *p =* 0.4	−0.0004 (−0.007 to 0.007); *p =* 0.9

a Estimates are shown with 95% confidence intervals for increase in atrophy rate in ml/year: for a doubling of WMH conditional on intracranial volume, CSF Aβ42 and tau.

b Estimates are shown with 95% confidence intervals for increase in atrophy rate in ml/year: for a 10 pg/ml increase in Aβ conditional on intracranial volume, log2WMH volume and CSF tau.

c Estimates are shown with 95% confidence intervals for increase in atrophy rate in ml/year: for a 10 pg/ml increase in tau, conditional on intracranial volume, Aβ42 and log2WMH volume.

d Estimates are shown with 95% confidence intervals for increase in atrophy rate in ml/year: for a doubling of WMH conditional on intracranial volume, CSF Aβ42 and tau, hippocampal atrophy rate.

Estimates are shown with 95% confidence intervals for increase in atrophy rate in ml/year: for a whole‐brain atrophy rate.

#### Associations between Aβ_42_ and atrophy rates adjusted for tau and log_2_WMH

In the CSF biomarker subset, after adjustment for tau and log_2_WMH, lower baseline CSF Aβ_42_ was associated with increased whole‐brain atrophy rates in controls with a 10 pg/ml decrease in concentration of Aβ_42_ associated with an increase in whole‐brain atrophy rate of 0.2 ml/year (0.06–0.3). In MCI patients lower Aβ_42_ was also associated with an increased whole‐brain atrophy rate; with a 10 pg/ml decrease in concentration of Aβ_42_ estimated to increase atrophy rate by 0.4 ml/year (0.2–0.6). In MCI patients evidence was observed for an association between Aβ_42_ and hippocampal atrophy, with a 10 pg/ml decrease in Aβ_42_ associated with an increase in atrophy rate of 0.006 ml/year (0.003–0.009).

#### Associations between CSF tau and atrophy rates adjusted for Aβ_42_ and log_2_WMH

In controls there was evidence that increasing CSF tau was associated with an increase in hippocampal atrophy rate adjusted for Aβ_42_ and log_2_WMH, with a 10 pg/ml increase in tau associated with an increase in atrophy rate of 0.005 ml/year (0.002–0.009). There were no other statistically significant associations.

### Associations Between log_2_WMH and Atrophy Rates Adjusted for Age and Vascular Risk Factors

Following age and VRF adjustment, WMH volume remained associated with hippocampal atrophy in controls and MCI patients, with a doubling of WMH burden associated with an increase in hippocampal atrophy rate of 0.004 ml/year (0.001–0.008) for controls and 0.007 ml/year (0.003–0.012) for MCI patients; although for controls this was not statistically significant following correction for concurrent whole‐brain atrophy, see Supporting Information Table 2. However, in models adjusting for CSF Aβ_42_, CSF tau, VRFs and age, WMH volume was found to be associated with hippocampal atrophy after adjusting for concurrent whole‐brain atrophy; with a doubling of WMH volume associated with an increase in atrophy rate of 0.005 ml/year (0.001–0.009) see Supporting Information Table 3.

### Association Between log_2_WMH and Atrophy Rate with a TIV‐WMH Interaction Term

Following the addition of an interaction term between WMH and TIV all significant associations of WMH to atrophy rate remained, see Supporting Information Table 4. The TIV‐WMH interaction term was significant for the whole‐brain of controls; at the mean TIV a doubling of WMH was found to be associated with an increase of 0.3 (0.07–0.51) ml/year in atrophy rate. This association between WMH and atrophy rate is then estimated to increase by 0.2 (0.01–0.38) ml/year for each 100 ml increase in TIV.

### VBM Results

Regions of reduced volume, or volume change, associated with WMH were observed in the precentral sulcus in all groups; the postcentral sulcus and superior frontal lobe for MCI and AD patients; and in the occipital lobes for control and MCI participants; see Table 5 for cluster locations, volumes, and *p* values, and Supporting Information for *t* and effects maps.

**Table 5:** Cross Sectional and Longitudinal VBM Results

		Cross‐sectional			Longitudinal		
		Location	*k*	*p*	Location	*k*	*p*
C	GM	Left precentral gyrus Left inferior temporal lobe	64 51	0.002 0.004	No results found		
	WM	No results found			Left superior frontal lobe	50	0.03
MCI	GM	Left parietal operculum	615	<0.001	Left occipital lobe	130	0.005
		Left thalamus, hypothalamus	865	<0.001	Right postcentral gyrus	53	0.009
		Right central sulcus	381	<0.001	Right occipital lobe	101	0.01
		Left central sulcus	191	0.001	Right superior frontal lobe	53	0.01
		Right parietal operculum	97	0.002	Right postcentral gyrus	54	0.01
					Right occipital	56	0.01
					Right postcentral gyrus	97	0.01
					Right cerebellum	232	0.02
					Right cerebellum	75	0.02
					Right superior frontal lobe	48	0.02
					Left superior frontal lobe	31	0.02
	WM	No results found			Right superior frontal lobe	1083	0.003
					Left superior frontal lobe	142	0.01
					Left precuneal	242	0.01
					Right precentral sulcus	116	0.02
					Right postcentral sulcus	80	0.02
AD	GM	Left occipital lobe	90	<0.001	No results found		
		Left occipital lobe	214	0.001			
		Right occipital lobe	284	0.001			
		Left central sulcus	42	0.003			
		Left hippocampus	303	0.004			
		Right central sulcus	47	0.005			
		Left occipital lobe	48	0.009			
	WM	No results found			No results found		

Results from voxel based morphometry (VBM) correlating log transformed white matter hyperintensity (log_2_WMH) with voxel volume adjusted for gender and total intracranial volume (cross sectional analysis), or log2WMH with change in voxel volume adjusted for TIV (longitudinal analysis). Tables show the cluster locations, size in number of voxels (*k*) and associated *p* value; analyses were corrected for multiple comparisons (Family Wise Error); clusters greater than 30 voxels and with a *P* value of >0.05 are reported. Table shows clusters in the grey matter and white matter for each control, MCI and AD subjects, from longitudinal and cross sectional VBM.

## DISCUSSION

In this study, we found novel evidence that WMHs were associated with disproportionately greater hippocampal atrophy in controls and MCI subjects relative to whole‐brain atrophy. Further, the associations in control subjects remain statistically significant when adjusting for CSF Aβ_42_ and tau in those subjects in whom CSF was taken (∼50% total cohort). In separate analyses for each structure we found a higher baseline WMH volume was associated with a greater atrophy rate of the whole‐brain and hippocampus, and of the hippocampus in MCIs. We also found evidence that increased WMH volume is associated with smaller cross‐sectional whole‐brain and hippocampal volumes across subject groups in ADNI1.

To our knowledge this is the first study to have reported an association between WMHs and longitudinal hippocampal loss occurring in cognitively normal older individuals after adjusting for AD pathology through measures of CSF Aβ_42_ and tau. In controls this observed relationship was equivalent to an 8.5% increase in mean hippocampal atrophy rate for a doubling of WMH volume in individuals with the average TIV, whole‐brain atrophy rate, and WMH burden. The disproportionate hippocampal loss observed may be due to the selective vulnerability of this region to WMHs or other shared risk factors for WMHs. Notably, the cause of such a relationship is not fully explained by AD pathology, at least as assessed by single CSF measurements, since relationships between atrophy and WMHs in normal individuals persist after adjustment for CSF Aβ_42_ and tau markers. As ADNI is a study designed to emulate a clinical trial, this is a finding of significant interest. The finding that WMHs are associated with atrophy independently of AD pathology (as measured by CSF biomarkers) mean a successful intervention targeting AD related pathology in controls with preclinical AD may not be as effective at slowing atrophy if there is concurrent white matter damage.

Associations between baseline WMHs and whole‐brain atrophy rate reached statistical significance only in controls and not in MCI subjects. This may reflect the inclusion criteria for ADNI to select amnestic presenting MCI patients, and for screening out those with significant vascular pathology. As a result those more likely to have predominantly AD pathology causing their cognitive impairment may have been selected. Further, this may be because WMHs have a greater association with structural changes early in the disease process, and a greater role for Aβ_42_ and tau in the early symptomatic AD stages.

Although cross‐sectional data indicates that the greatest association between WMHs and atrophy is in the AD group, longitudinal data show no evidence of an association between baseline WMH volume and subsequent atrophy in AD subjects. Longitudinal relationships between WMH and atrophy rates were detected in controls. A possible explanation for these observations is that WMHs exert a similar influence on atrophy rates in control and AD subjects but that this is difficult to detect in the AD group due to large inter‐subject heterogeneity and lower power due to smaller group size. Factors causing variance in atrophy rates in AD are yet to be fully determined but may include inflammatory factors, disease duration and genetics. Alternatively WMHs may have a primary influence on progressive atrophy early in the disease course and less at later stages. A notable caveat of this latter interpretation is that these similarly aged diagnostic groups may not represent a continuum across the disease spectrum; a 75‐year‐old control with a high WMH burden destined to develop AD may follow a different disease course from a 75‐year‐old with established disease. Larger group sizes and continuation of longitudinal data collection over longer periods will provide more information to address the nature of the relationship between WMHs and atrophy rates in the presence and absence of AD.

Our results are in line with previous studies showing that WMHs are associated with reduced grey matter volume (Rossi et al., 2006; Wen et al., 2006; Raji et al., 2012; Lambert et al., 2015) and progressive atrophy (Enzinger et al., 2005a; Barnes et al., 2013). However, unlike Rossi et al. and Raji et al., we did not find volume loss associated with WMH volume in the prefrontal cortex in our VBM analyses (Rossi et al., 2006; Raji et al., 2012). Other studies have also found smaller hippocampal volumes in subjects with higher WMH volumes (de Leeuw et al., 2004; den Heijer et al., 2005), whilst others using longitudinal measures of hippocampal atrophy have not (Du et al., 2006; Nosheny et al., 2015). In a recent study Nosheny et al. did not find a relationship between WMH and hippocampal atrophy rate in control or MCI subjects using multivariable models, despite also using ADNI1 subjects. This key difference may be due to the use of subjects with longitudinal MRI and also either CSF Aβ_42_, or amyloid positron emission tomography (PET) imaging data collected at any point over a 4‐year period, including the transition in phase to ADNIGO or ADNI2 (Nosheny et al., 2015). This length of follow‐up may be important; in our study we have found that those who have at least a 24‐month scan have higher brain and lower WMH volume than subjects who drop out before 24 months (see Supporting Information data). Alternatively, our findings may be due to our inclusion of tau as a covariate as well as Aβ_42_, or use of BSI rather than Freesurfer methods; hippocampal measures have been previously been found to be highly variable between techniques, likely due to differing definitions of the region (Cash et al., 2015).Our study extends these findings by showing that greater longitudinal hippocampal atrophy is seen in control subjects with a higher burden of WMHs, after adjusting for concurrent brain atrophy and CSF biomarkers.

That statistically significant relationships between atrophy rates and WMHs are predominantly seen in control subjects is in agreement with previous longitudinal studies showing changes in WM before grey matter changes occur, observed both at the macroscopic level through acceleration in growth of WMHs (Silbert et al. 2012) and microscopically through changes in water diffusion in WM detected by diffusion tensor imaging (DTI) (Zhuang et al., 2012). This is in concordance with the two hit vascular hypothesis for AD; that vascular risk factors may confer blood‐brain‐barrier dysfunction and oligaemia in the first instance, initiating a second cascade of events involving amyloid and tau, neuronal dysfunction, degeneration, cognitive decline and eventual dementia (Zlokovic, 2011).

The observed hippocampal atrophy may be driven by an independent direct effect of WMH, and/or by vascular risk factors and ageing processes which also cause WMH. Notably, associations between the hippocampal atrophy and WMH remained in controls following adjustment for VRFs and age in our analyses, suggesting WMH may have an effect on atrophy rate over and above the influence of the effects of the VRFs investigated in this study. Specific vulnerability of the hippocampus to WMH was suggested by the fact that an association was still observed after adjusting for concurrent whole‐brain atrophy, CSF biomarkers, age and VRFs.

The dual vulnerability of the hippocampus to vascular and AD pathology is congruent with studies demonstrating the susceptibility of the hippocampus to hypertension in midlife (Korf et al., 2004) and hypoxia (Kirino and Sano, 1984; Di Paola et al., 2008; Horstmann et al., 2010). Alternatively WMH may contribute to atrophy through cortical disconnection (Villain et al., 2008; Lee et al., 2010) and/or WMHs in tracts serving the hippocampus may lead to axonal loss and subsequent atrophy via Wallerian degeneration (Von Bohlen und Halbach and Unsicker, 2002; Schmidt et al., 2011). Whether the observed link between hippocampal atrophy and WMH is dependent on TIV (a prodromal marker of brain size) is yet unknown and presents an interesting question for future research.

We have found that the relationship between WMH volume and atrophy persists when adjusting for CSF biomarkers of AD pathology in controls. Whilst a separate vascular means of tissue loss is plausible, Aβ_42_, tau, and WMHs may be intimately related. There is evidence from animal models that following ischaemia there is an acute increase in secretions of Aβ (Pluta et al., 2013), and following resuscitation from cardiac arrest in humans, Zetterberg et al. found a time‐dependent increase in Aβ_42_ levels detected in the blood (Zetterberg et al., 2011). Recent work has shown that MCI participants with low tau, but higher regional WMHs, display increased entorhinal cortex atrophy (Tosto et al., 2015). Finally, poor vascular health may contribute to Aβ_42_ and tau deposition due to impaired clearance of toxins from the brain via the perivascular drainage pathways (Tarasoff‐Conway et al., 2015).

Our VBM analyses did not find a longitudinal association between WMHs and atrophy in the hippocampal region but did find significant positive associations in other cortical regions. That we did not find statistically significant evidence of an association between hippocampal atrophy rate and WMHs in these analyses may be due to the lower WMH burden and slower progression in the subset with a usable baseline and 24 month scan pair (see Supporting Information Table 1), or of the difficulty of this technique at detecting specific hippocampal change. Those with unusable 24‐month scans had lower brain volumes and higher WMH volumes at baseline than those who had good quality 24‐month imaging. Other studies have found such selection biases: Leung et al. found, within a similar subset of ADNI, a trend that suggested controls and AD subjects with higher brain atrophy rates between baseline and 12 months would be less likely to have an available 12 to 24 month scan pair (Leung et al., 2013). This suggests our VBM findings may be biased toward those with a less aggressive disease course and less white matter disease. In addition, VBM was performed on a single (baseline–24 months) scan pair and BSIs on all available scan pairs including those who dropped out before 24 months, thus reducing power with which to detect an association with VBM.

VBM analyses showed subjects with higher WMH volume had lower cross‐sectional volumes in somatosensory and motor cortices in control and subjects, and greater longitudinal change in MCI patients. These regions are among the last areas to be affected by amyloid plaques and neuritic tangles in AD (Brun and Englund, 1981; Braak and Braak, 1991), suggesting tissue loss in these regions is more likely to be related to shared vascular processes underlying WMH burden than AD pathology. Atrophy in motor regions related to WMHs is also supported by clinical symptoms of impaired gait and motor control experienced by individuals with a high burden of WMHs, as reported previously (Longstreth et al., 1996; Whitman et al., 2001; Silbert et al., 2008). Gait disturbance may be caused by involvement of periventricular WMHs disrupting descending and ascending sensory and motor pathways (Longstreth et al., 1996).

Limitations of this study are that there are biases in the study sample which may limit generalisability to a wider population. These associations were found in healthy ADNI controls who were free from overt cardiovascular disease (subjects with a Hachinski score ≤4 included), were well educated and of a high socioeconomic status. As such these findings may be different compared to a community population, who may have a greater vascular burden. The imaging data for this set of ADNI did not acquire T2‐weighted fluid attenuated inversion recovery (FLAIR) MRI which gives a more accurate estimation of white matter hyperintensity volume. Our measures may not be optimal as it is becoming clearer that novel techniques such as diffusion MRI can detect subtle white matter changes before they become visible on standard structural MRI (de Groot et al., 2013). Additionally we looked at a global, total measure of WMHs and did not look at subtypes of WMH, such as deep or periventricular, nor did we split by regions, such as frontal or parietal WMHs; these may have given differing results (Tosto et al., 2015). Lastly, to fully investigate the impact of small vessel disease on atrophy rates, further markers of small vessel disease, such as lacunes and microbleeds, and larger subject sizes allowing the unpicking of relationships between vascular covariates, imaging markers of vascular damage and atrophy would be required.

In summary, our study indicates that WMH load is associated with hippocampal atrophy in healthy older adults after adjusting for CSF measures of AD pathology. These results show that baseline WMH can partially explain variability in atrophy rates; therefore clinical trials to target AD pathology in at‐risk populations may increase power with which to detect a treatment effect by stratifying or adjusting for WMH. Further longitudinal investigation will be required to determine whether WMH are an important step on the pathway to atrophy and cognitive impairment. If they are, subsequent preventative approaches designed to reduce the risk of developing WMH could therefore potentially decrease the incidence or delay the onset of atrophy and cognitive impairment.

## Supporting information

Supporting Information

## References

[hipo22690-bib-0001] Ashburner J , Ridgway GR. 2013 Symmetric diffeomorphic modeling of longitudinal structural MRI. Front Neurosci 6:197. 23386806 10.3389/fnins.2012.00197 PMC3564017

[hipo22690-bib-0002] Barnes J , Carmichael OT , Leung KK , Schwarz C , Ridgway GR , Bartlett JW , Malone IB , Schott JM , Rossor MN , Biessels GJ , DeCarli C , Fox NC. 2013 Vascular and Alzheimer's disease markers independently predict brain atrophy rate in Alzheimer's Disease Neuroimaging Initiative controls. Neurobiol Aging 34:1996–2002. 23522844 10.1016/j.neurobiolaging.2013.02.003 PMC3810644

[hipo22690-bib-0003] Von Bohlen und Halbach O , Unsicker K. 2002 Morphological alterations in the amygdala and hippocampus of mice during ageing'. Eur J Neurosci 16:2434–2440. 12492438 10.1046/j.1460-9568.2002.02405.x

[hipo22690-bib-0004] Braak H , Braak E. 1991 Neuropathological stageing of Alzheimer‐related changes. Acta Neuropathol 82:239–259. 1759558 10.1007/BF00308809

[hipo22690-bib-0005] Brun A , Englund E. 1981 Regional pattern of degeneration in Alzheimer's disease: Neuronal loss and histopathological grading. Histopathology 5:549–564. 7286917 10.1111/j.1365-2559.1981.tb01818.x

[hipo22690-bib-0006] Carmichael O , Schwarz C , Drucker D , Fletcher E , Harvey D , Beckett L , Jack CR , Weiner M , DeCarli C. 2010 Longitudinal changes in white matter disease and cognition in the first year of the Alzheimer disease neuroimaging initiative. Arch Neurol 67:1370–1378. 21060014 10.1001/archneurol.2010.284 PMC3082636

[hipo22690-bib-0007] Cash DM , Frost C , Iheme LO , Ünay D , Kandemir M , Fripp J , Salvado O , Bourgeat P , Reuter M , Fischl B , Lorenzi M , Frisoni GB , Pennec X , Pierson RK , Gunter JL , Senjem ML , Jack CR , Guizard N , Fonov VS , Collins DL , Modat M , Cardoso MJ , Leung KK , Wang H , Das SR , Yushkevich PA , Malone IB , Fox NC , Schott JM , Ourselin S. 2015 Assessing atrophy measurement techniques in dementia: Results from the MIRIAD atrophy challenge'. NeuroImage 123:149–164. 26275383 10.1016/j.neuroimage.2015.07.087 PMC4634338

[hipo22690-bib-0008] Crane DE , Black SE , Ganda A , Mikulis DJ , Nestor SM , Donahue MJ , MacIntosh BJ. 2015 matter blood flow and volume are reduced in association with white matter hyperintensity lesion burden: A cross‐sectional MRI study'. Front Aging Neurosci 7:1–8. 26217223 10.3389/fnagi.2015.00131 PMC4495336

[hipo22690-bib-0009] Debette S , Seshadri S , Beiser A , Au R , Himali JJ , Palumbo C , Wolf PA , DeCarli C. 2011 Midlife vascular risk factor exposure accelerates structural brain aging and cognitive decline. Neurology 77:461–468. 21810696 10.1212/WNL.0b013e318227b227 PMC3146307

[hipo22690-bib-0010] Du AT , Schuff N , Chao LL , Kornak J , Jagust WJ , Kramer JH , Reed BR , Miller BL , Norman D , Chui HC , Weiner MW. 2006 Age effects on atrophy rates of entorhinal cortex and hippocampus. Neurobiol Aging 27:733–740. 15961190 10.1016/j.neurobiolaging.2005.03.021 PMC1779763

[hipo22690-bib-0011] Dubois B , Feldman HH , Jacova C , Hampel H , Molinuevo JL , Blennow K , DeKosky ST , Gauthier S , Selkoe D , Bateman R , Cappa S. 2014 Advancing research diagnostic criteria for Alzheimer's disease: The IWG‐2 criteria. Lancet Neurol 13:614–629. p 24849862 10.1016/S1474-4422(14)70090-0

[hipo22690-bib-0012] Eckerström C , Olsson E , Klasson N , Bjerke M , Göthlin M , Jonsson M , Rolstad S , Malmgren H , Wallin A , Edman Å. 2011 High white matter lesion load is associated with hippocampal atrophy in mild cognitive impairment. Dementia Geriatr Cognit Disord 31:132–138. 10.1159/000323014 21293123

[hipo22690-bib-0013] Enzinger C , Fazekas F , Matthews PM , Ropele S , Schmidt H , Smith S , Schmidt R. 2005 Risk factors for progression of brain atrophy in aging: Six‐year follow‐up of normal subjects. Neurology 64:1704–1711. 15911795 10.1212/01.WNL.0000161871.83614.BB

[hipo22690-bib-0014] Fernando MS , Ince PG. 2004 Vascular pathologies and cognition in a population‐based cohort of elderly people. J Neurol Sci 226:13–17. 15537512 10.1016/j.jns.2004.09.004

[hipo22690-bib-0015] van der Flier WM , van der Vlies AE , Weverling‐Rijnsburger AWE , de Boer NL , Admiraal‐Behloul F , Bollen ELEM , Westendorp RGJ , van Buchem MA , Middelkoop HAM . 2005 MRI measures and progression of cognitive decline in nondemented elderly attending a memory clinic. Int J Geriatr Psychiatry 20:1060–1066. 16250078 10.1002/gps.1392

[hipo22690-bib-0016] Fox NC , Scahill RI , Crum WR , Rossor MN. 1999 Correlation between rates of brain atrophy and cognitive decline in AD. Neurology 52:1687–1689. 10331700 10.1212/wnl.52.8.1687

[hipo22690-bib-0017] Gattringer T , Enzinger C , Ropele S , Gorani F , Petrovic KE , Schmidt R , Fazekas F. 2012 Vascular risk factors, white matter hyperintensities and hippocampal volume in normal elderly individuals. Dementia Geriatr Cognit Disord 33:29–34. 10.1159/000336052 22377559

[hipo22690-bib-0018] de Groot M , Verhaaren BFJ , de Boer R , Klein S , Hofman A , van der Lugt A , Ikram MA , Niessen WJ , Vernooij MW. 2013 Changes in normal‐appearing white matter precede development of white matter lesions. Stroke 44:1037–1042. 23429507 10.1161/STROKEAHA.112.680223

[hipo22690-bib-0019] Hampel H , Bürger K , Pruessner JC , Zinkowski R , DeBernardis J , Kerkman D , Leinsinger G , Evans AC , Davies P , Möller HJ , Teipel SJ. 2005 Correlation of cerebrospinal fluid levels of tau protein phosphorylated at threonine 231 with rates of hippocampal atrophy in Alzheimer disease. Arch Neurol 62:770–773. 15883264 10.1001/archneur.62.5.770

[hipo22690-bib-0020] den Heijer T , Launer LJ , Prins ND , van Dijk EJ , Vermeer SE , Hofman a , Koudstaal PJ , Breteler MMB. 2005 Association between blood pressure, white matter lesions, and atrophy of the medial temporal lobe. Neurology 64:263–267. 15668423 10.1212/01.WNL.0000149641.55751.2E

[hipo22690-bib-0021] Henneman WJP , Sluimer JD , Barnes J , van der Flier WM , Sluimer IC , Fox NC , Scheltens P , Vrenken H , Barkhof F. 2009 Hippocampal atrophy rates in Alzheimer disease'. Neurology 72:999–1007. 19289740 10.1212/01.wnl.0000344568.09360.31 PMC2821835

[hipo22690-bib-0022] Horstmann A , Frisch S , Jentzsch RT , Muller K , Villringer A , Schroeter ML. 2010 Resuscitating the heart but losing the brain: Brain atrophy in the aftermath of cardiac arrest. Neurology 74:306–312. 20101036 10.1212/WNL.0b013e3181cbcd6f

[hipo22690-bib-0023] Jack CR , Bernstein MA , Fox NC , Thompson P , Alexander G , Harvey D , Borowski B , Britson PJ , Whitwell JL , Ward C , Dale AM , Felmlee JP , Gunter JL , Hill DLG , Killiany R , Schuff N , Fox‐bosetti S , Lin C , Studholme C , Charles S , Krueger G , Ward HA , Metzger GJ. 2008 The Alzheimer's disease neuroimaging initiative (ADNI): MRI methods. J Magn Reson 27:685–691. 10.1002/jmri.21049 PMC2544629 18302232

[hipo22690-bib-0024] Jack CR , Petersen RC , Xu Y , O'Brien PC , Smith GE , Ivnik RJ , Boeve BF , Tangalos EG , Kokmen E. 2000 Rates of hippocampal atrophy correlate with change in clinical status in aging and AD. Neurology 55:484–490. 10953178 10.1212/wnl.55.4.484 PMC2724764

[hipo22690-bib-0025] Jack CR , Shiung MM , Weigand SD , O'Brien PC , Gunter JL , Boeve BF , Knopman DS , Smith GE , Ivnik RJ , Tangalos EG , Petersen RC. 2005 Brain atrophy rates predict subsequent clinical conversion in normal elderly and amnestic MCI. Neurology 65:1227–1231. 16247049 10.1212/01.wnl.0000180958.22678.91 PMC2753547

[hipo22690-bib-0026] Jellinger KA , Attems J. 2007 Neuropathological evaluation of mixed dementia. J Neurol Sci 257:80–87. 17324442 10.1016/j.jns.2007.01.045

[hipo22690-bib-0027] Jovicich J , Czanner S , Greve D , Haley E , Van Der Kouwe A , Gollub R , Kennedy D , Schmitt F , Brown G , MacFall J , Fischl B , Dale A. 2006 Reliability in multi‐site structural MRI studies: Effects of gradient non‐linearity correction on phantom and human data. NeuroImage 30:436–443. 16300968 10.1016/j.neuroimage.2005.09.046

[hipo22690-bib-0028] Kirino T , Sano K. 1984 Selective vulnerability in the gerbil hippocampus following transient ischemia. Acta Neuropathol 62:201–208. 6695554 10.1007/BF00691853

[hipo22690-bib-0029] Knopman DS , Griswold ME , Lirette ST , Gottesman RF , Kantarci K , Sharrett AR , Jack CR , Graff‐Radford J , Schneider ALC , Windham BG , Coker LH , Albert MS , Mosley TH , Coresh J , Roche KB , Selnes OA , McKhann G , Alonso A , Folsom AR , Eckfeldt J , Wagenknecht LE , Heiss G , Couper D , Wruck L. 2015 Vascular Imaging abnormalities and cognition: Mediation by cortical volume in nondemented individuals: Atherosclerosis risk in communities‐neurocognitive study. Stroke 46:433–440. 25563642 10.1161/STROKEAHA.114.007847 PMC4308430

[hipo22690-bib-0030] Korf ESC , White LR , Scheltens P , Launer LJ. 2004 Midlife blood pressure and the risk of hippocampal atrophy: The Honolulu Asia aging study. Hypertension 44:29–34. 15159381 10.1161/01.HYP.0000132475.32317.bb

[hipo22690-bib-0031] Lambert C , Narean JS , Benjamin P , Zeestraten E , Barrick TR , Markus HS. 2015 Characterising the grey matter correlates of Leukoaraiosis in cerebral small vessel disease. NeuroImage Clincal 9:194–205. 10.1016/j.nicl.2015.07.002 PMC4564392 26448913

[hipo22690-bib-0032] Lee DY , Fletcher E , Martinez O , Zozulya N , Kim J , Tran J , Buonocore M , Carmichael O , Decarli C. 2010 Vascular and degenerative processes differentially affect regional interhemispheric connections in normal aging, mild cognitive impairment, and Alzheimer disease. Stroke 41:1791–1797. 20595668 10.1161/STROKEAHA.110.582163 PMC2922914

[hipo22690-bib-0033] de Leeuw FE , Barkhof F , Scheltens P. 2004 White matter lesions and hippocampal atrophy in Alzheimer's disease. Neurology 62:310–312. 14745078 10.1212/01.wnl.0000103289.03648.ad

[hipo22690-bib-0034] Leung KK , Barnes J , Modat M , Ridgway GR , Bartlett JW , Fox NC , Ourselin S. 2011 Brain MAPS: An automated, accurate and robust brain extraction technique using a template library. NeuroImage 55:1091–1108. 10.1016/j.neuroimage.2010.12.067 PMC3554789 21195780

[hipo22690-bib-0035] Leung KK , Barnes J , Ridgway GR , Bartlett JW , Clarkson MJ , Macdonald K , Schuff N , Fox NC , Ourselin S. 2010 Automated cross‐sectional and longitudinal hippocampal volume measurement in mild cognitive impairment and Alzheimer's disease. NeuroImage 51:1345–1359. 20230901 10.1016/j.neuroimage.2010.03.018 PMC2873209

[hipo22690-bib-0036] Leung KK , Bartlett JW , Barnes J , Manning EN , Ourselin S , Fox NC. 2013 Cerebral atrophy in mild cognitive impairment and Alzheimer disease: Rates and acceleration'. Neurology 80:648–654. 23303849 10.1212/WNL.0b013e318281ccd3 PMC3590059

[hipo22690-bib-0037] Leung KK , Ridgway GR , Ourselin S , Fox NC. 2012 Consistent multi‐time‐point brain atrophy estimation from the boundary shift integral. NeuroImage 59:3995–4005. 22056457 10.1016/j.neuroimage.2011.10.068

[hipo22690-bib-0038] Longstreth W , Manolio T , Arnold A. 1996 Clinical correlates of white matter findings on cranial magnetic resonance imaging of 3301 elderly people: The Cardiovascular Health Study. Stroke 1274–1282. 8711786 10.1161/01.str.27.8.1274

[hipo22690-bib-0039] Malone IB , Leung KK , Clegg S , Barnes J , Whitwell JL , Ashburner J , Fox NC , Ridgway GR. 2015 Accurate automatic estimation of total intracranial volume: A nuisance variable with less nuisance. NeuroImage 104:366–372. 25255942 10.1016/j.neuroimage.2014.09.034 PMC4265726

[hipo22690-bib-0040] Manning EN , Barnes J , Cash DM , Bartlett JW , Leung KK , Ourselin S , Fox NC. 2014 APOE ε4 is associated with disproportionate progressive hippocampal atrophy in AD. PLoS One 9:e97608. 24878738 10.1371/journal.pone.0097608 PMC4039513

[hipo22690-bib-0041] McEvoy LK , Edland SD , Holland D , Hagler DJ Jr, Roddey JC , Fennema‐Notestine C , Salmon DP , Koyama AK , Aisen PS , Brewer JB , Dale AM. 2010 Neuroimaging enrichment strategy for secondary prevention trials in Alzheimer's disease. Alzheimer Dis Assoc Disord 24 p 269. 20683184 10.1097/WAD.0b013e3181d1b814 PMC2929320

[hipo22690-bib-0042] McKhann G , Drachman D , Folstein M , Katzman R , Price D , Stadlan EM. 1984 Clinical diagnosis of Alzheimer's disease Report of the NINCDS‐ADRDA Work Group* under the auspices of Department of Health and Human Services Task Force on Alzheimer's Disease. Neurology 34:939–939. 6610841 10.1212/wnl.34.7.939

[hipo22690-bib-0043] Mungas D , Reed BR , Jagust WJ , DeCarli C , Mack WJ , Kramer JH , Weiner MW , Schuff N , Chui HC. 2002 Volumetric MRI predicts rate of cognitive decline related to AD and cerebrovascular disease. Neurology 59:867–873. 12297568 10.1212/wnl.59.6.867 PMC1820873

[hipo22690-bib-0044] Narayana P. a , Brey WW , Kulkarni MV , Sievenpiper CL. 1988 Compensation for surface coil sensitivity variation in magnetic resonance imaging. Magn Reson Imaging 6:271–274. 3398733 10.1016/0730-725x(88)90401-8

[hipo22690-bib-0045] Nosheny RL , Insel PS , Truran D , Schuff N , Jack CR , Aisen PS , Shaw LM , Trojanowski JQ , Weiner MW. 2015 Variables associated with hippocampal atrophy rate in normal aging and mild cognitive impairment. Neurobiol Aging 36:273–282. 25175807 10.1016/j.neurobiolaging.2014.07.036 PMC5832349

[hipo22690-bib-0046] Ota M , Nemoto K , Sato N , Mizukami K , Yamashita F , Asada T. 2011 Relationship between white matter T2 hyperintensity and cortical volume changes on magnetic resonance imaging in healthy elders. Int J Geriatr Psychiatry 26:886–892. 20872420 10.1002/gps.2618

[hipo22690-bib-0047] Di Paola M , Caltagirone C , Fadda L , Sabatini U , Serra L , Carlesimo GA. 2008 Hippocampal atrophy is the critical brain change in patients with hypoxic amnesia'. Hippocampus 18:719–728. 18446831 10.1002/hipo.20432

[hipo22690-bib-0048] Pluta R , Furmaga‐Jabłońska W , MacIejewski R , Ułamek‐Kozioł M , Jabłoński M. 2013 Brain ischemia activates β‐ and γ‐secretase cleavage of amyloid precursor protein: Significance in sporadic Alzheimer's disease. Mol Neurobiol 47:425–434. 23080191 10.1007/s12035-012-8360-z PMC3538125

[hipo22690-bib-0049] Van De Pol LA , Van Der Flier WM , Korf ESC , Fox NC , Barkhof F , Scheltens P. 2007 Baseline predictors of rates of hippocampal atrophy in mild cognitive impairment. Neurology 69:1491–1497. 17923611 10.1212/01.wnl.0000277458.26846.96

[hipo22690-bib-0050] Prins ND , van Straaten ECW , van Dijk EJ , Simoni M , van Schijndel RA , Vrooman HA , Koudstaal PJ , Scheltens P , Breteler MMB , Barkhof F. 2004 Measuring progression of cerebral white matter lesions on MRI: Visual rating and volumetrics. Neurology 62:1533–1539. 15136677 10.1212/01.wnl.0000123264.40498.b6

[hipo22690-bib-0051] Raji C. a , Lopez OL , Kuller LH , Carmichael OT , Longstreth WT , Gach HM , Boardman J , Bernick CB , Thompson PM , Becker JT. 2012 White matter lesions and brain gray matter volume in cognitively normal elders. Neurobiol Aging 33:834–e7. 10.1016/j.neurobiolaging.2011.08.010 PMC3248984 21943959

[hipo22690-bib-0052] Ridgway GR , Omar R , Ourselin S , Hill DLG , Warren JD , Fox NC. 2009 Issues with threshold masking in voxel‐based morphometry of atrophied brains. NeuroImage 44:99–111. 18848632 10.1016/j.neuroimage.2008.08.045

[hipo22690-bib-0053] Román GC , Tatemichi TK , Erkinjuntti T , Cummings JL , Masdeu JC , Garcia JH , Amaducci L , Orgogozo JM , Brun A , Hofman A. 1993 Vascular dementia: diagnostic criteria for research studies. Report of the NINDS‐AIREN International Workshop. Neurology 43:250–250. 8094895 10.1212/wnl.43.2.250

[hipo22690-bib-0054] Rossi R , Boccardi M , Sabattoli F , Galluzzi S , Alaimo G , Testa C , Frisoni GB. 2006 Topographic correspondence between white matter hyperintensities and brain atrophy. J Neurol 253:919–927. 16502217 10.1007/s00415-006-0133-z

[hipo22690-bib-0055] Schmidt P , Gaser C , Arsic M , Buck D , Förschler A , Berthele A , Hoshi M , Ilg R , Schmid VJ , Zimmer C , Hemmer B , Mühlau M. 2012 An automated tool for detection of FLAIR‐hyperintense white‐matter lesions in multiple sclerosis. NeuroImage 59:3774–3783. 22119648 10.1016/j.neuroimage.2011.11.032

[hipo22690-bib-0056] Schmidt R , Ropele S , Enzinger C , Petrovic K , Smith S , Schmidt H , Matthews PM , Fazekas F. 2005 White matter lesion progression, brain atrophy, and cognitive decline: The Austrian stroke prevention study. Ann Neurol 58:610–616. 16178017 10.1002/ana.20630

[hipo22690-bib-0057] Schmidt R , Schmidt H , Haybaeck J , Loitfelder M , Weis S , Cavalieri M , Seiler S , Enzinger C , Ropele S , Erkinjuntti T , Pantoni L , Scheltens P , Fazekas F , Jellinger K. 2011 Heterogeneity in age‐related white matter changes. Acta Neuropathol 122:171–185. 21706175 10.1007/s00401-011-0851-x

[hipo22690-bib-0058] Schneider J. a , Arvanitakis Z , Bang W , Bennett DA 2007 Mixed brain pathologies account for most dementia cases in community‐dwelling older persons. Neurology 69:2197–2204. 17568013 10.1212/01.wnl.0000271090.28148.24

[hipo22690-bib-0059] Schuff N , Woerner N , Boreta L , Kornfield T , Shaw LM , Trojanowski JQ , Thompson PM , Jack CR , Weiner MW. 2009 MRI of hippocampal volume loss in early Alzheimer's disease in relation to ApoE genotype and biomarkers. Brain 132:1067–1077. 19251758 10.1093/brain/awp007 PMC2668943

[hipo22690-bib-0060] Schwarz C , Fletcher E , Decarli C , Carmichael O. 2009 Fully‐automated white matter hyperintensity detection with anatomical prior knowledge and without FLAIR. In: Lecture Notes in Computer Science (Including Subseries Lecture Notes in Artificial Intelligence and Lecture Notes in Bioinformatics). 5636 LNCS. pp 239–251, Springer Berlin Heidelberg. 10.1007/978-3-642-02498-6_20 PMC2864489 19694267

[hipo22690-bib-0061] Shaw LM , Vanderstichele H , Knapik‐czajka M , Clark CM , Aisen PS , Petersen RC , Blennow K , Soares H , Simon A , Lewczuk P , Dean R , Siemers E , Potter W , Lee VM , Trojanowski JQ. 2009 Cerebrospinal fluid biomarker signature in Alzheimer's disease neuroimaging initiative subjects. Ann Neurol 65:403–413. 19296504 10.1002/ana.21610 PMC2696350

[hipo22690-bib-0062] Silbert LC , Dodge HH , Perkins LG , Sherbakov L , Lahna D , Erten‐Lyons D , Woltjer R , Shinto L , Kaye JA. 2012 Trajectory of white matter hyperintensity burden preceding mild cognitive impairment. Neurology 79:741–747. 22843262 10.1212/WNL.0b013e3182661f2b PMC3421153

[hipo22690-bib-0063] Silbert LC , Nelson C , Howieson DB , Moore MM , Kaye JA. 2008 Impact of white matter hyperintensity volume progression on rate of cognitive and motor decline. Neurology 71:108–113. 18606964 10.1212/01.wnl.0000316799.86917.37 PMC2676966

[hipo22690-bib-0064] Sled JG , Zijdenbos AP , Evans AC. 1998 A nonparametric method for automatic correction of intensity nonuniformity in MRI data. IEEE Trans Med Imaging 17:87–97. 9617910 10.1109/42.668698

[hipo22690-bib-0065] Tarasoff‐Conway JM , Carare RO , Osorio RS , Glodzik L , Butler T , Fieremans E , Axel L , Rusinek H , Nicholson C , Zlokovic BV , Frangione B , Blennow K , Ménard J , Zetterberg H , Wisniewski T , de Leon MJ. 2015 Clearance systems in the brain—Implications for Alzheimer disease. Nat Rev Neurol 11:248. 26195256 10.1038/nrneurol.2015.119 PMC4694579

[hipo22690-bib-0066] Tosto G , Zimmerman ME , Hamilton JL , Carmichael OT , Brickman AM , Alzheimer's Disease Neuroimaging Initiative 2015 The effect of white matter hyperintensities on neurodegeneration in mild cognitive impairment. Alzheimer Dementia 11:1510–1519. 10.1016/j.jalz.2015.05.014 PMC4677059 26079417

[hipo22690-bib-0067] Villain N , Desgranges B , Viader F , de la Sayette V , Mézenge F , Landeau B , Baron JC , Eustache F , Chételat G. 2008 Relationships between hippocampal atrophy, white matter disruption, and gray matter hypometabolism in Alzheimer's disease. J Neurosci 28:6174–6181. 18550759 10.1523/JNEUROSCI.1392-08.2008 PMC2902815

[hipo22690-bib-0068] Wen W , Sachdev PS , Chen X , Anstey K. 2006 Gray matter reduction is correlated with white matter hyperintensity volume: A voxel‐based morphometric study in a large epidemiological sample. NeuroImage 29:1031–1039. 10.1016/j.neuroimage.2005.08.057 16253521

[hipo22690-bib-0069] Whitman GT , Tang Y , Lin a , Baloh RW. 2001 A prospective study of cerebral white matter abnormalities in older people with gait dysfunction. Neurology 57:990–994. 11571322 10.1212/wnl.57.6.990

[hipo22690-bib-0070] Ye BS , Seo SW , Kim GH , Noh Y , Cho H , Yoon CW , Kim HJ , Chin J , Jeon S , Lee JM , Seong J‐K , Kim JS , Lee J‐H , Choe YS , Lee KH , Sohn YH , Ewers M , Weiner M , Na DL. 2014 Amyloid burden, cerebrovascular disease, brain atrophy, and cognition in cognitively impaired patients. Alzheimer Dementia 11:494–503. 10.1016/j.jalz.2014.04.521 PMC5862069 25048578

[hipo22690-bib-0071] Yoshita Fletcher E , Harvey D , Ortega M , Martinez O , Mungas DM , Reed BR , DeCarli CS. 2006 Extent and distribution of white matter hyperintensities in normal aging, MCI, and AD. Neurology 67:2192–2198. 17190943 10.1212/01.wnl.0000249119.95747.1f PMC3776588

[hipo22690-bib-0072] Yu P , Sun J , Wolz R , Stephenson D , Brewer J , Fox NC , Cole PE , Jack CR , Hill DLG , Schwarz AJ. 2014 Operationalizing hippocampal volume as an enrichment biomarker for amnestic mild cognitive impairment trials: Effect of algorithm, test‐retest variability, and cut point on trial cost, duration, and sample size. Neurobiol Aging 35:808–818. 24211008 10.1016/j.neurobiolaging.2013.09.039 PMC4201941

[hipo22690-bib-0073] Zetterberg H , Mörtberg E , Song L , Chang L , Provuncher GK , Patel PP , Ferrell E , Fournier DR , Kan CW , Campbell TG , Meyer R , Rivnak AJ , Pink BA , Minnehan KA , Piech T , Rissin DM , Duffy DC , Rubertsson S , Wilson DH , Blennow K. 2011 Hypoxia due to cardiac arrest induces a time‐dependent increase in serum amyloid β levels in humans. PLoS One 6: e28263. 22194817 10.1371/journal.pone.0028263 PMC3237426

[hipo22690-bib-0074] Zhuang L , Sachdev PS , Trollor JN , Kochan NA , Reppermund S , Brodaty H , Wen W. 2012 Microstructural white matter changes in cognitively normal individuals at risk of amnestic MCI. Neurology 79:748–754. 22843270 10.1212/WNL.0b013e3182661f4d

[hipo22690-bib-0075] Zlokovic BV. 2011 Neurovascular pathways to neurodegeneration in Alzheimer's disease and other disorders. Nat Rev Neurosci 12:723–738. 22048062 10.1038/nrn3114 PMC4036520

