# Supplementary material for: White matter hyperintensities are associated with disproportionate progressive hippocampal atrophy
Source: Hippocampus. 2017 Jan 9;27(3):249–62. doi: 10.1002/hipo.22690 (PMC5324634; doi:10.1002/hipo.22690)
Supplement: Supplementary file 1 — Supporting Information [file HIPO-27-249-s001.docx]

**Supplementary Material: White matter hyperintensities are associated with disproportionate hippocampal atrophy**

Contents

[Supplementary Methods 1](#_Toc441661427)

[Supplementary Results 2](#_Toc441661433)

[Supplementary Figures 5](#_Toc441661438)

References 7

# Supplementary Methods

### Demographics

Subjects with baseline and 24 month scans were used in the VBM section of this study. As such this VBM group represents a subset of subjects used in the main BSI analyses (456 of 697): those who remained in the study to the 24 month stage and had a good quality baseline and 24 month repeat scan. To examine whether there is any bias between the VBM subgroup and those in which VBM was not performed (due to drop out or poor quality scans), differences in demographics were explored. Linear regressions were performed for continuous variables (age, MMSE, total brain volume, total hippocampal volume, TIV and WMH) with VBM membership entered as a categorical predictor in the model. To investigate gender differences between groups a logistic regression was used with predictors of VBM group membership and diagnostic group and outcome of gender.

### Cross-Sectional VBM

SPM 12b was used for all VBM analyses in MATLAB version R2012a. Baseline T1-weighted volumetric scans were rigidly reoriented using an in-house script. Briefly, images were affinely registered in SPM to MNI space using mutual information, the rigid transformation extracted using Procrustes Decomposition and applied to the image transformation matrix (images were not resliced). The reoriented images were assessed to ensure proper alignment. Images were segmented using SPM12’s unified segmentation, with 2 Gaussian components for each of GM, WM and CSF, with other settings left at default values. DARTEL was performed to nonlinearly register images and to create a group specific space based upon simultaneous alignment of GM and WM. GM and WM segments were warped to DARTEL space, preserving tissue volume, and smoothed with a kernel of 6mm.

Masks were created for GM and WM separately, using the smoothed, modulated, warped segments (Ridgway et al., 2009). The masking technique averages the segments to be masked and creates a mask based on thresholding that average, which maximises the correlation between the original segment and the thresholded segment.

### Longitudinal VBM

24 month T1-weighted volumetric scans used for BSI analyses were selected. Scans were reoriented to MNI space, to facilitate quality assurance, as before. Using SPM12’s pairwise longitudinal registration tool, baseline and 24 month scans were registered (Ashburner and Ridgway, 2012). This registration produces an unbiased average image, corresponding to the midpoint between the two time points. Images of the rate of volumetric voxel expansion and contraction were produced, given by the difference between the Jacobian determinant maps for each time-point (which encode each voxel’s volume in the baseline and 24 month images relative to the midpoint image) divided by the time interval. These volume change maps were visually assessed, as were the midpoint averages. The midpoint averages were segmented and registered with DARTEL, as described for the baselines above. Bias corrected midpoint averages, GM, WM segments and volume change maps from the pairwise registration were then DARTEL transformed. During this step inputs were not modulated (values rather than volumes were preserved). DARTEL transformed bias corrected midpoint average images for all subjects were then averaged in order to create an image with which to overlay any results.

Tissue weighted smoothing (also known as normalised convolution) was applied using the DARTEL transformed GM and volume change maps, with a kernel of 6mm. This step smooths the volume change maps using only data within the limits of the tissue segments (binarised at 0.5). The result is a tissue-specific smoothed volume change map (each voxel value corresponding to expansion or contraction of the particular tissue during longitudinal registration). This was repeated for WM. The smoothed GM and WM tissue weighted volume change maps were then used for longitudinal analysis.

### Statistical Analysis

A flexible factorial model was used to investigate the relationship between log_2_WMH volume at baseline and voxel volume (or change in volume) in each of the diagnostic groups. Interaction terms were used between group and log_2_WMH, and t-contrasts used to investigate relationships between WMH and volume in each diagnostic group. Both the contrasts in the expected direction and reverse contrasts were run.

Resultant maps of t-values were overlaid on the group average image and thresholded at p<0.05 following correction for multiple comparisons using family wise error correction (FWE). Partial correlation coefficient maps were additionally generated to assess the strength and directionality of all associations at each voxel using a colour scale. The following equation was used to calculate this, r = t /√ (t^2^ + df), where r is the partial correlation coefficient, t is the t value and df the degrees of freedom.

### CSF subset VBM

Additional longitudinal VBM analyses were run on the CSF subgroup. VBM steps were completed as per the pipeline described above until the DARTEL stage, in which the DARTEL registration was limited to the subset of the VBM group with CSF data (n= 249). The volume change maps, grey and white matter segments of the subset were aligned to this CSF specific template. The volume change maps were smoothed as above. Additionally CSF subset specific GM and WM masks were created as before. Flexible factorial models were used as described above, t-contrasts were used to assess relationships between WMH and longitudinal change in each group after controlling for biomarkers which were entered into the design matrix as covariates. Three models were run, with CSF Aβ1-42 as a covariate, tau as a covariate and with both Aβ1-42 and tau as covariates. Results were thresholded at p<0.05 following FWE correction, and viewed as before.

# Supplementary Results

### Demographics

Supplementary table 1 shows demographic and imaging summary statistics for the group used for VBM and BSI analyses and those who were used in BSI analyses alone (selected based on presence of useable 24 month scan). Of note, the VBM subset had significantly larger whole-brain volumes adjusted for TIV, and significantly lower WMH volumes at baseline adjusted for TIV. Additionally, MCI and AD subjects used for both BSI and VBM had very similar brain volumes, but not after adjusting for TIV proportionally.

## Longitudinal models adjusted for age and vascular risk factors

Supplementary table 2 shows the results of longitudinal models of WMH volume and atrophy rate after adjustment for vascular risk factors (VRFs) and age. In these models WMH was associated with increased hippocampal atrophy rate in controls and MCI subjects of 0.004 ml/year (0.001, 0.008) for controls and 0.007 ml/year (0.003, 0.012) for MCI patients. WMH was also associated with whole brain atrophy in MCI patients, a doubling of which related to an increase in whole brain atrophy of 0.35 ml/year (0.07 – 0.6). The relationship between WMH and hippocampal atrophy rate was not statistically significant following adjustment for concurrent whole brain atrophy.

In analyses adjusted for age, VRFs and CSF biomarkers abeta and tau (supplementary table 3), WMH was not associated with whole brain or hippocampal atrophy in MCI patients. In control patients increasing WMH was associated with an increase in whole brain and hippocampal atrophy, with a doubling of WMH predicting an increase in whole brain atrophy rate of 0.34 ml/year (0.10 - 0.58) and hippocampal atrophy rate of 0.007 ml/year (0.003, 0.001). The relationship between WMH and hippocampal atrophy rate was significant after adjustment for concurrent whole brain atrophy rate; with a doubling of WMH associated with an increase in hippocampal atrophy rate of 0.005 ml/year (0.001, 0.009).

## Longitudinal models with a WMH-TIV interaction term

Supplementary table 4 shows the results of the model including a TIV-WMH interaction term. The interaction term was significant for the whole brain of controls; at the mean TIV a doubling of WMH was found to be associated with an increase of 0.3 ml/year in atrophy rate. This association between WMH and atrophy rate is then estimated to increase by 0.2 ml/year for each 100ml increase in TIV. The overall association of WMH to whole brain atrophy remained in this group.

All statistically significant overall associations of WMH to hippocampal and whole-brain atrophy rate remained as before across subject groups, see table 4. This was tested by comparing models with TIV-WMH interaction and with WMH as predictors, versus models without these coefficients using a likelihood ratio test.

### Cross-Sectional VBM

#### Baseline Grey Matter

VBM analyses showed cross-sectional associations between higher WMH and lower regional brain volumes in control subjects (see supplementary figure 1) with significant clusters in the left medial temporal gyrus, right thalamic nuclei and right central sulcus. Correlation coefficient maps show the positive (red) and negative (blue) correlations between WMH and volume, which indicate the non-significant association in hippocampal regions with WMH. In MCI patients, WMH was associated with significantly lower volumes bilaterally in the pre- and post- central gyrus, parietal operculum, thalamus and hypothalamus. Additionally in the MCI group increased WMH volume was associated with lower volumes in right anterior cingulate, orbitofrontal cortex and left insula. In ADs greater WMH was associated with significant clusters in bilateral occipital lobes, left hippocampus, left cuneus, midbrain, and the right posterior temporal lobe along the medial surface. As evidenced by the effect maps, WMH was associated with smaller hippocampi bilaterally, but this reached significance in the left hippocampus only. Due to the highly atrophied brains in this group (see supplementary table 1), it is possible that the clusters following the interface between CSF and brain may reflect partial volume.

#### Baseline White Matter

There were no relationships between WMH and reduced white matter volume in any subject group. With a reverse contrast a 5 voxel cluster in the left orbitofrontal cortex in was present in controls, representing a greater volume with greater WMH volume. This is an area highly susceptible to artefact, therefore likely does not reflect a true effect, see supplementary figure 3.

### Longitudinal VBM

#### Longitudinal Grey Matter Change

Longitudinally, greater WMH levels were associated with increased progressive grey matter atrophy in the cingulate region in controls, see effect map supplementary figure 2, with a single voxel surviving FWE correction in the right hemisphere. Effect maps indicate a relationship between WMH and atrophy in hippocampal regions which does not reach significance in controls and MCI subjects. In MCIs greater WMH volume was significantly associated with tissue loss of the bilateral occipital lobes, superior frontal lobes and the right pre- and post- central gyrus and cerebellum. In cerebellar and central sulcal regions the association between WMH, GM and WM atrophy survived correction only on the right side; inspection of effect maps shows symmetry also on the left side. There were no relationships between GM atrophy and WMH in ADs.

#### Longitudinal White Matter Change

Longitudinal contraction of the left superior frontal WM was related to greater WMH level in controls, see supplementary figure 2. In MCIs increased WMH volume was associated with white matter atrophy of the bilateral superior frontal lobe, cuneus, left cingulate gyrus and of the right postcentral, precentral and supramarginal gyrus. There were no associations between WMH and WM atrophy in ADs. With a reverse contrast, a growth in WM at the lining of the ventricles was associated with higher WMH in ADs; this likely represents expansion of WMH as this is a susceptible area to white matter disease, see supplementary figure 3.

### CSF Subset

Longitudinal VBM on subset with CSF data (n= 249) revealed no significant clusters in any group.

|  | Subjects used in VBM and BSI analyses | | | Subjects used in BSI analyses alone | | | P value across two subgroups |
| --- | --- | --- | --- | --- | --- | --- | --- |
|  | **Controls** | **MCI** | **AD** | **Controls** | **MCI** | **AD** |  |
| N (% of subgroup) | 150 (33) | 217 (48) | 89 (19) | 48 (20) | 128 (53) | 65 (27) |  |
| Age at baseline, years | 75.9 (4.9) | 74.9 (7.0) | 75.4 (7.0) | 76.2 (5.9) | 75.1 (7.6) | 74.5 (8.4) | 0.8 |
| Percentage male | 50.7 | 63.9 | 51.7 | 58.3 | 62.0 | 56.9 | 0.6 |
| MMSE at baseline, /30 | 29.2 (1.0) | 27.1 (1.8) | 23.2 (1.9) | 28.9 (1.2) | 26.7 (1.7) | 23.7 (1.9) | 0.4* |
| Total brain volume, ml | 1069.6 (102.8) | 1069.5(111.4) | 1022.6 (118.7) | 1063.5 (103.9) | 1045.4 (118.6) | 1021.4 (110.8) | 0.02** |
| Total hippocampal volume, ml | 5.2 (0.8) | 4.5 (0.8) | 3.9 (1.0) | 5. 1 (0.6) | 4.4 (0.9) | 4.0 (0.81) | 0.4** |
| Total intracranial volume, ml | 1445 (133) | 1475 (141) | 1441 (169) | 1446.5 (139.6) | 1455.0 (151.9) | 1460.7 (165.2) | 0.8 |
| WMH, ml  log_2_WMH, ml | 0.22 (0.41)  -2.38 (2.3) | 0.23 (0.48)  -2.37 (2.5) | 0.36 (1.00)  -1.35 (2.1) | 0.25 (0.60)  -2.32 (2.5) | 0.34 (0.65)  -1.59 (2.1) | 0.44 (0.61)  -1.38 (2.3) | 0.02** |

# Supplementary Figures

#

Supplementary table 1: Mean subject demographic information and basic imaging information for the subset used for both VBM and BSI analyses and those used in the BSI analyses alone (without good quality 24 month scans) *adjusted for diagnostic group **and additionally TIV. WMH values reported as median, with IQR, tests of significance applied to WMH on log scale.

|  | **Controls** | **MCI** | **AD** |
| --- | --- | --- | --- |
| **N** | 198 | 345 | 154 |
| **Rate of whole brain atrophy ml/year** | 5.93  (<0.01)  [5.06, 6.80] | 9.11  (<0.01)  [7.84,10.37] | 13.49  (<0.01)  [11.02, 15.95] |
| **Rate of hippocampal atrophy ml/year** | 0.06  (<0.01)  [0.04, 0.07] | 0.11  (<0.01)  [0.09, 0.13] | 0.17  (<0.01)  [0.13, 0.20] |
| **Association between WMH and whole brain atrophy^a^** | 0.213  (0.06)  [-0.007, 0.434] | 0.346  (0.02)  [0.066, 0.627] | 0.187  (0.45)  [-0.295, 0.668] |
| **Association between WMH and hippocampal atrophy^a^** | 0.004  (0.02)  [0.001, 0.008] | 0.007  (<0.01)  [0.003, 0.012] | -0.002  (0.55)  [-0.009, 0.005] |
| **Association between WMH and whole brain atrophy rate adjusted for hippocampal atrophy rate^b^** | 0.062  (0.5)  [-0.121, 0.246] | 0.023  (0.8)  [-0.188, 0.233] | 0.270  (0.2)  [-0.131, 0.670] |
| **Association between WMH and hippocampal atrophy rate adjusted for whole brain atrophy rate^c^** | 0.002  (0.12)  [-0.001, 0.005] | 0.004  (0.03)  [0.001, 0.007] | -0.004  (0.2)  [-0.009, 0.002] |

Supplementary Table 2: Results from the regression models assessing the relationship between atrophy rates (outcome measures) and log_2_WMH volume (predictor). Estimates are shown for increase in atrophy rate (ml/year), with 95% confidence intervals: for a doubling of WMH, adjusted for intracranial volume, history of smoking, hyperlipidaemia, diabetes, hypertension, APOE genotype, and age^a^; additionally hippocampal atrophy rate^b^ ; or whole brain atrophy rate^c^ .

Supplementary table 3: Results from the regression models assessing the relationship between atrophy rates (outcome measures) and log_2_WMH volume, CSF Aβ and tau (predictors) all adjusted for adjusted for intracranial volume, history of smoking, hyperlipidaemia, diabetes, hypertension, APOE genotype, and age (covariates). Estimates are shown with 95% confidence intervals for an increase in atrophy rate in ml/year: for a doubling of WMH adjusted for CSF Aβ and tau^a^, for a 10 pg/ml increase in Aβ conditional on log_2_WMH volume and CSF tau^b^, for a 10 pg/ml increase in tau, adjusted for Aβ and log_2_WMH volume^c^ and for a doubling of WMH conditional on intracranial volume, CSF Aβ and tau and hippocampal atrophy rate^d^ or whole brain atrophy rate^e^

|  | **Controls** | **MCI** | **AD** |
| --- | --- | --- | --- |
| **N** | 100 | 167 | 86 |
| **Rate of whole brain atrophy (ml/year)** | 5.28  (<0.01)  [4.27, 6.29] | 10.69  (<0.01)  [8.85, 12.53] | 14.73  (<0.01)  [11.10, 18.36] |
| **Rate of hippocampal atrophy (ml/year)** | 0.05  (<0.01)  [0.03, 0.07] | 0.12  (<0.01)  [0.09, 0.15] | 0.18  (<0.01)  [0.13, 0.23] |
| **Association between WMH and whole brain atrophy rate^a^** | 0.336  (0.01)  [0.095, 0.578] | 0.332  (0.10)  [-0.066, 0.729] | -0.127  (0.65)  [-0.686, 0.431] |
| **Association between WMH and hippocampal atrophy rate^a^** | 0.007  (<0.01)  [0.003, 0.012] | 0.005  (0.12)  [-0.001, 0.011] | -0.003  (0.40)  [-0.011, 0.004] |
| **Association between CSF Aβ and whole brain atrophy rate^b^** | -0.180  (<0.01)  [-0.306, -0.055] | -0.289  (<0.01)  [-0.488, -0.091] | -0.194  (0.27)  [-0.542, 0.154] |
| **Association between CSF Aβ and hippocampal atrophy rate^b^** | -0.002  (0.06)  [-0.004, 0.000] | -0.005  (<0.01)  [-0.008, -0.002] | -0.003  (0.15)  [-0.008, 0.001] |
| **Association between CSF tau and whole brain atrophy rate^c^** | 0.315  (0.01)  [0.093, 0.537] | 0.047  (0.66)  [-0.161, 0.255] | 0.047  (0.71)  [-0.201, 0.296] |
| **Association between CSF tau and hippocampal atrophy rate^c^** | 0.006  (<0.01)  [0.002, 0.010] | 0.001  (0.57)  [-0.002, 0.004] | -0.001  (0.65)  [-0.004, 0.003] |
| **Association between WMH and whole brain atrophy rate adjusted for hippocampal atrophy rate^d^** | 0.160  (0.2)  [-0.083, 0.404] | 0.121  (0.4)  [-0.177, 0.419] | -0.006  (1.0)  [-0.500, 0.487] |
| **Association between WMH and hippocampal atrophy rate adjusted for whole brain atrophy rate^e^** | 0.005  (<0.01)  [0.001, 0.009] | 0.001  (0.6)  [-0.003, 0.006] | -0.002  (0.4)  [-0.009, 0.004] |

Supplementary table 3: Results from the regression models assessing the relationship between atrophy rates (outcome measures) and log_2_WMH volume, CSF Aβ and tau (predictors) all adjusted intracranial volume, history of smoking, hyperlipidaemia, diabetes, hypertension, APOE genotype, and age (covariates). Estimates are shown with 95% confidence intervals for an increase in atrophy rate in ml/year: for a doubling of WMH adjusted for CSF Aβ and tau^a^, for a 10 pg/ml increase in Aβ conditional on log_2_WMH volume and CSF tau^b^, for a 10 pg/ml increase in tau, adjusted for Aβ and log_2_WMH volume^c^ and for a doubling of WMH conditional on intracranial volume, CSF Aβ and tau and hippocampal atrophy rate^d^ or whole brain atrophy rate^e^

Supplementary table 3: Results from the regression models assessing the relationship between atrophy rates (outcome measures) and log_2_WMH volume, CSF Aβ and tau (predictors) all adjusted for adjusted for intracranial volume, history of smoking, hyperlipidaemia, diabetes, hypertension, APOE genotype, and age (covariates). Estimates are shown with 95% confidence intervals for an increase in atrophy rate in ml/year: for a doubling of WMH adjusted for CSF Aβ and tau^a^, for a 10 pg/ml increase in Aβ conditional on log_2_WMH volume and CSF tau^b^, for a 10 pg/ml increase in tau, adjusted for Aβ and log_2_WMH volume^c^ and for a doubling of WMH conditional on intracranial volume, CSF Aβ and tau and hippocampal atrophy rate^d^ or whole brain atrophy rate^e^

|  | **Controls** | **MCI** | **AD** |
| --- | --- | --- | --- |
| **N** | 100 | 167 | 86 |
| Rate of whole brain atrophy (ml/year) | 6.24  (p<0.01)  [5.75 - 6.72] | 10.68  (p<0.01)  [10.00 - 11.37] | 15.10  (p<0.01)  [14.03 - 16.16] |
| Rate of hippocampal atrophy (ml/year) | 0.06  (p<0.01)  [0.06 - 0.07] | 0.14  (p<0.01)  [0.13 - 0.15] | 0.20  (p<0.01)  [0.19 - 0.22] |
| Association between WMH and whole brain atrophy rate^a^ | 0.291  (0.01)  [0.073 - 0.509] | 0.118  (0.41)  [-0.164 - 0.399] | 0.044  (0.86)  [-0.458 - 0.546] |
| Association between WMH and hippocampal atrophy rate^a^ | 0.006  (p<0.01)  [0.002 - 0.009] | 0.005  (0.03)  [0.001 - 0.009] | -0.001  (0.88)  [-0.008 - 0.007] |
| Association between WMH-TIV interaction term and whole brain atrophy rate^b^ | 0.195  (0.04)  [0.012 - 0.377] | 0.075  (0.44)  [-0.114 - 0.264] | -0.036  (0.83)  [-0.360 - 0.288] |
| Association between WMH-TIV interaction term and hippocampal atrophy rate^b^ | 0.002  (0.21)  [-0.001 - 0.005] | 0.002  (0.17)  [-0.001 - 0.005] | -0.000  (0.93)  [-0.005 - 0.004] |
| Overall test of association of WMH to brain atrophy rate† | p=0.01 | p=0.55 | p=0.95 |
| Overall test of association of WMH to hippocampal atrophy rate† | p=0.01 | p=0.04 | p=0.99 |

Supplementary Table 4: Results from the regression models assessing the relationship between atrophy rates (outcome measures) and log_2_WMH volume, log_2_WMH-TIV interaction and TIV (predictors). Estimates are shown for increase in atrophy rate (ml/year), with 95% confidence intervals: for a doubling of WMH^a^, or for a 100ml increase in TIV^b^. †p value for the overall association of WMH to atrophy rate.

**Controls**

**MCI**

Grey Matter

**L ––––––––––––––––––––––––––––––––––––––––––––––––––––––––> R**

**P –––––––––––––––––––––––––––––––––––––––––––> A**


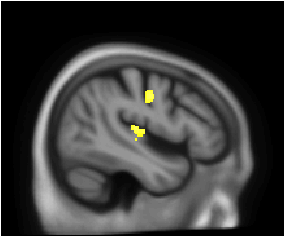

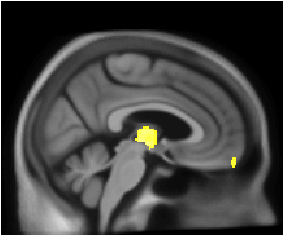

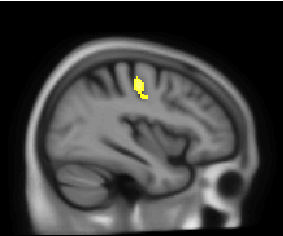


**Alzheimer’s**

**disease**


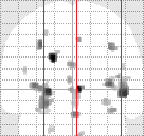

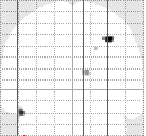

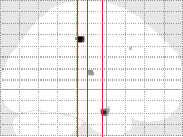

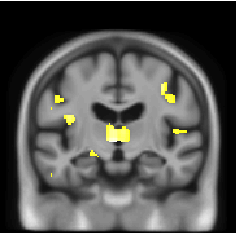

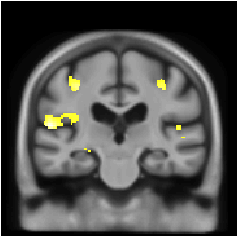

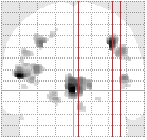

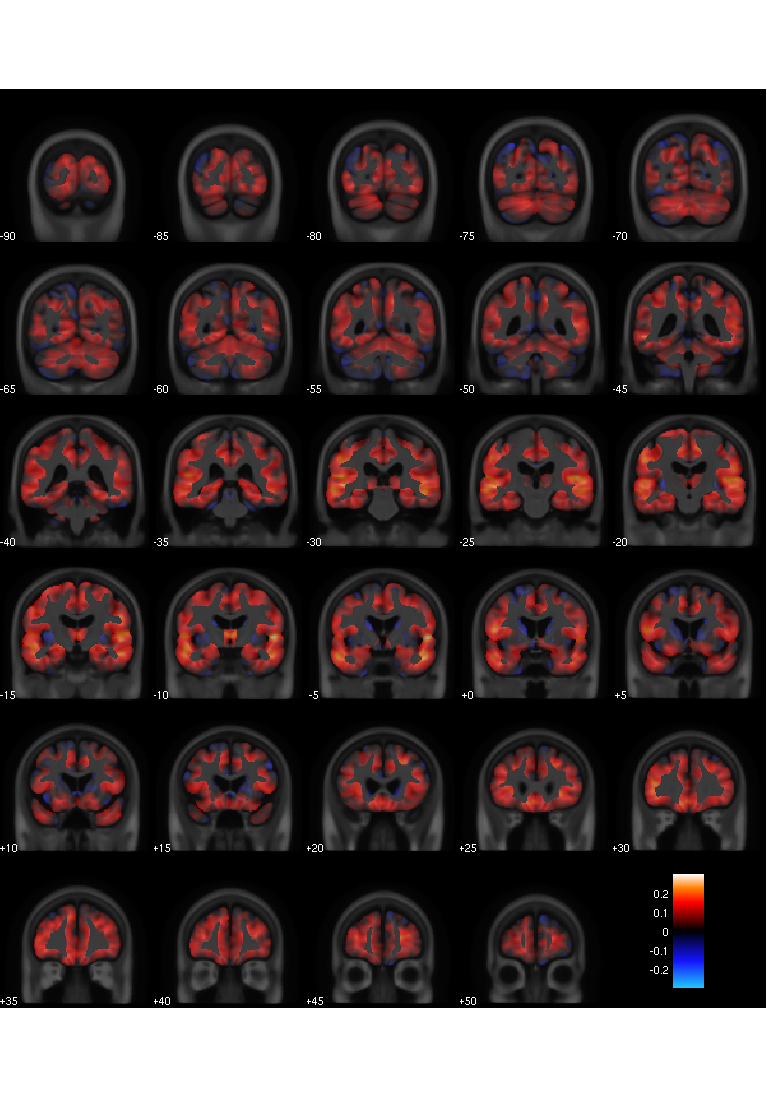

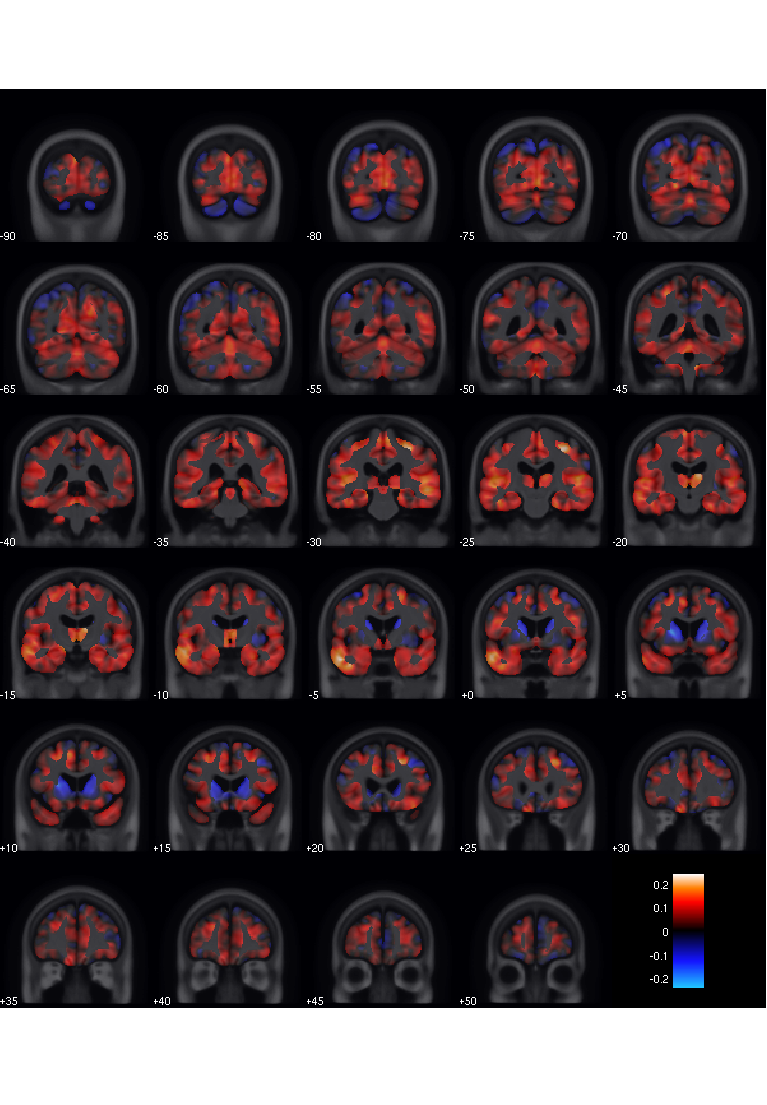

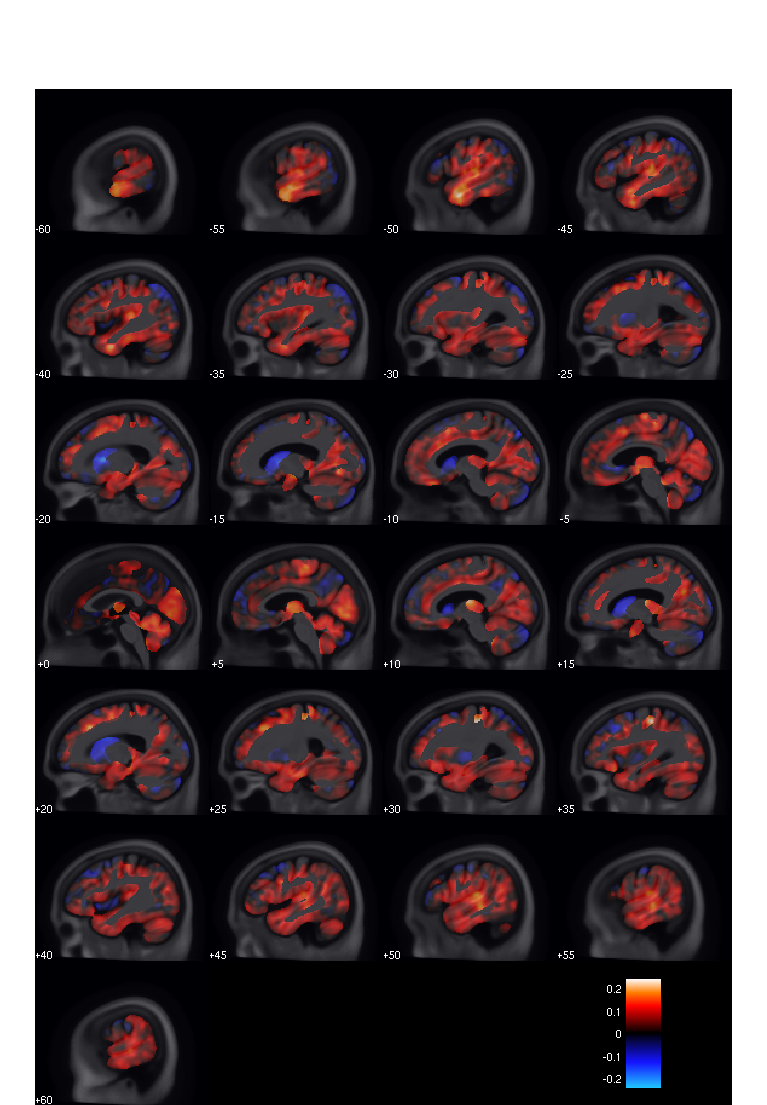

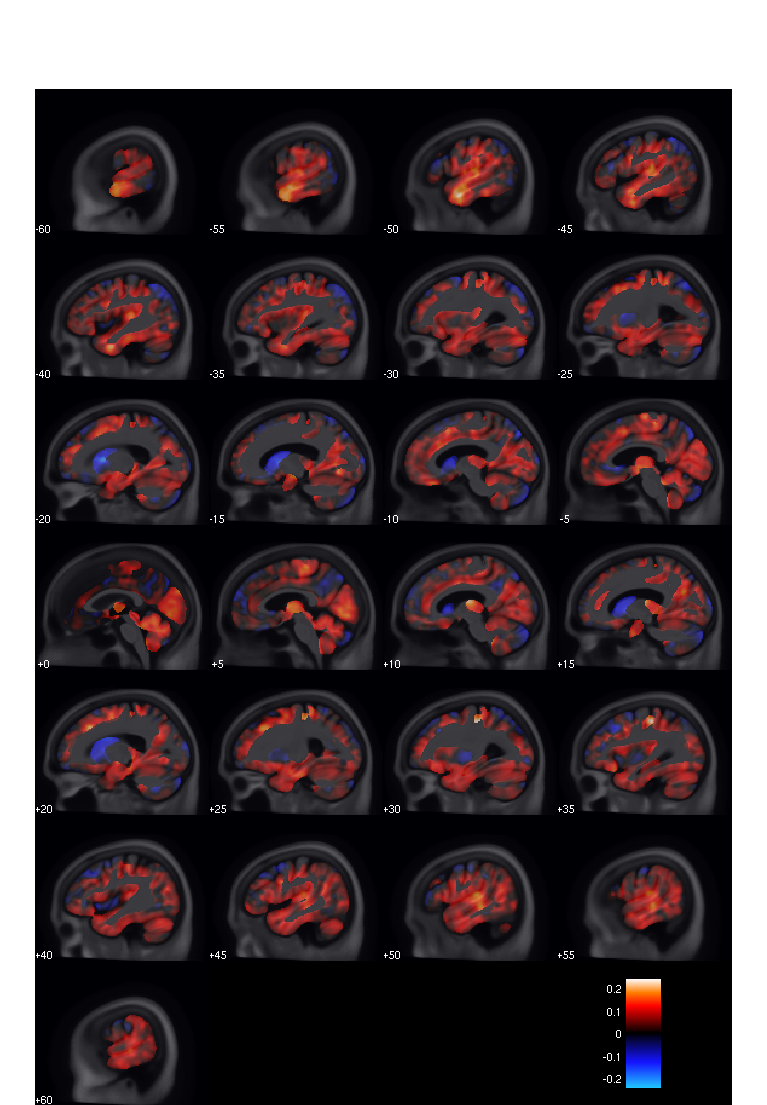

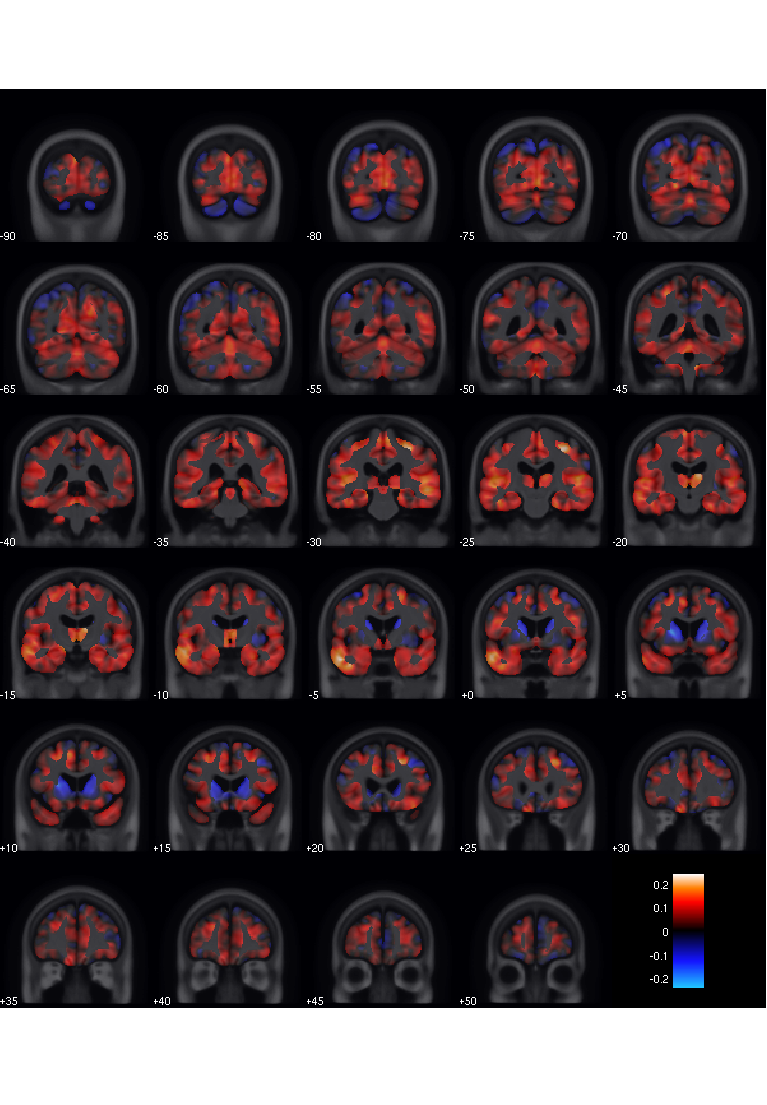

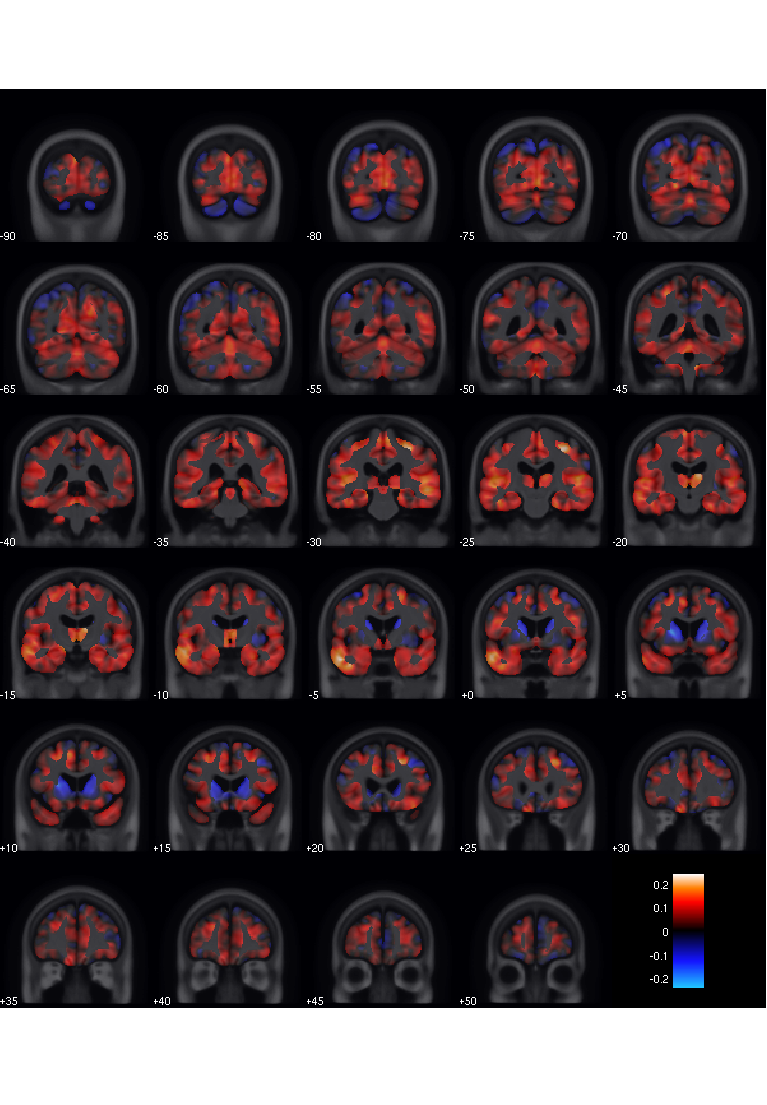

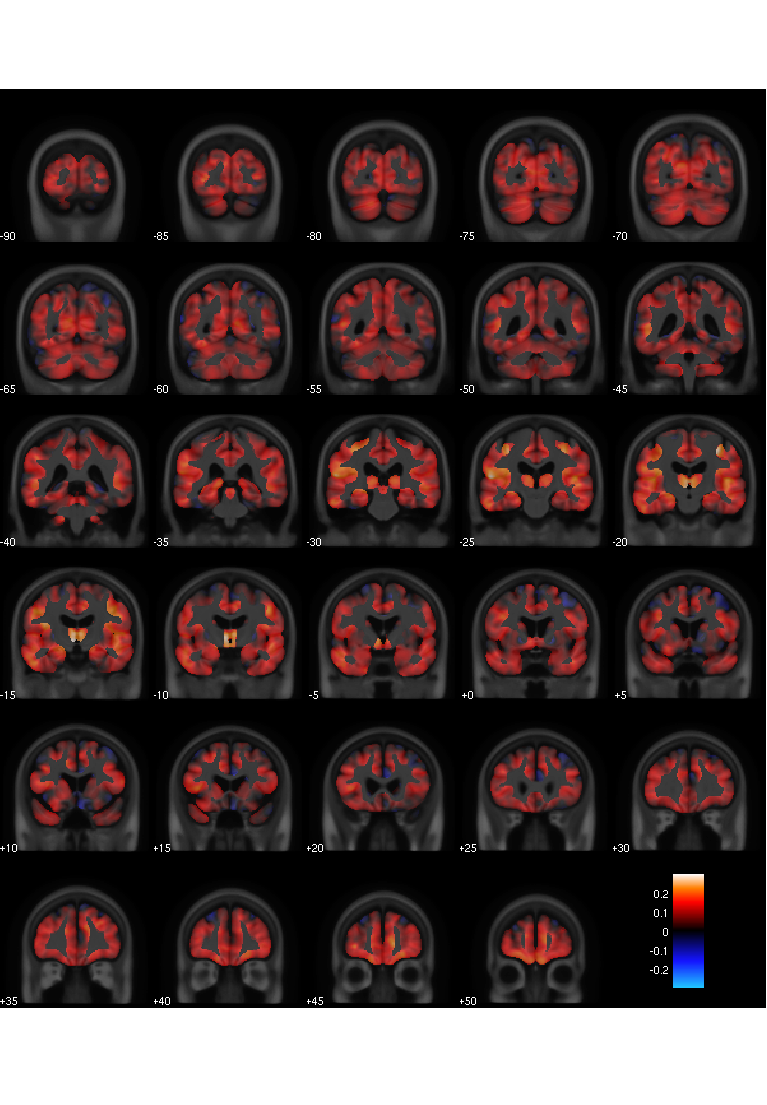

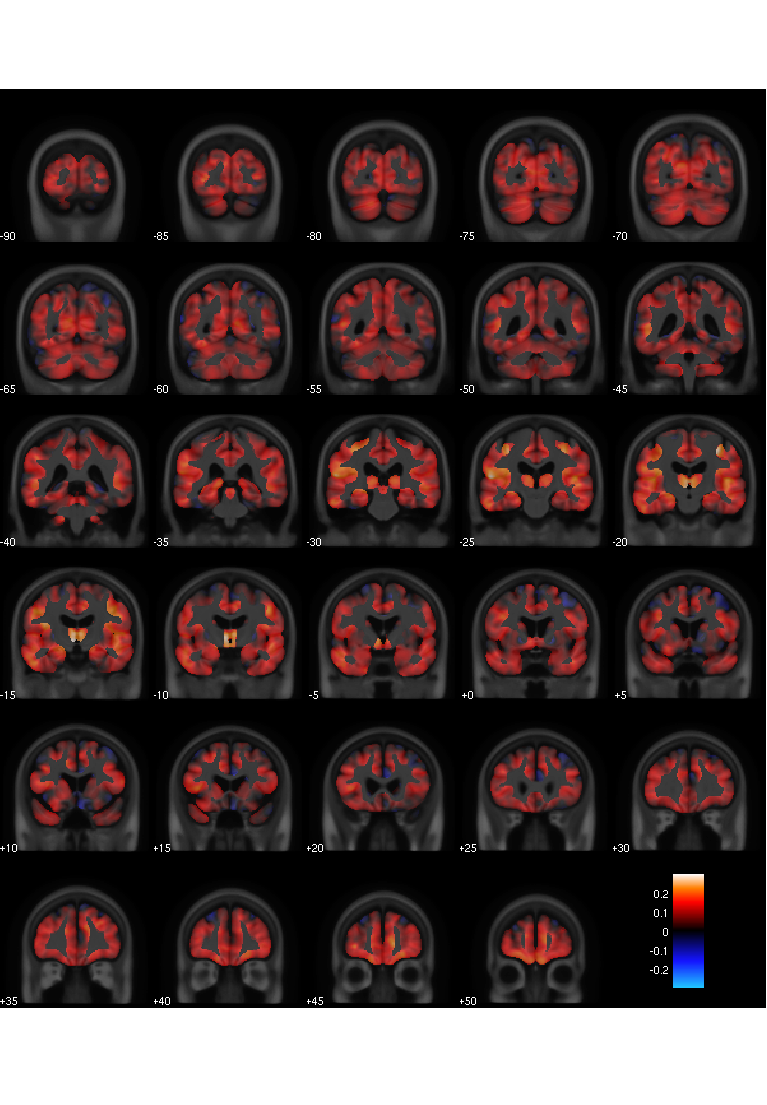

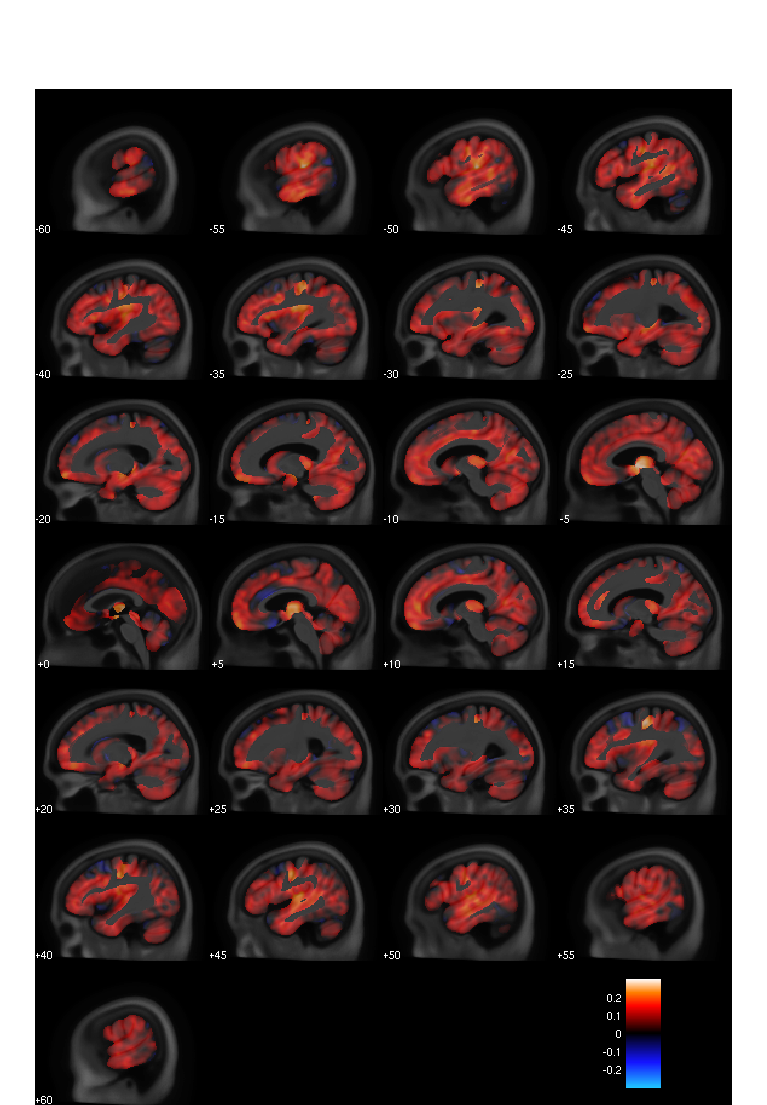

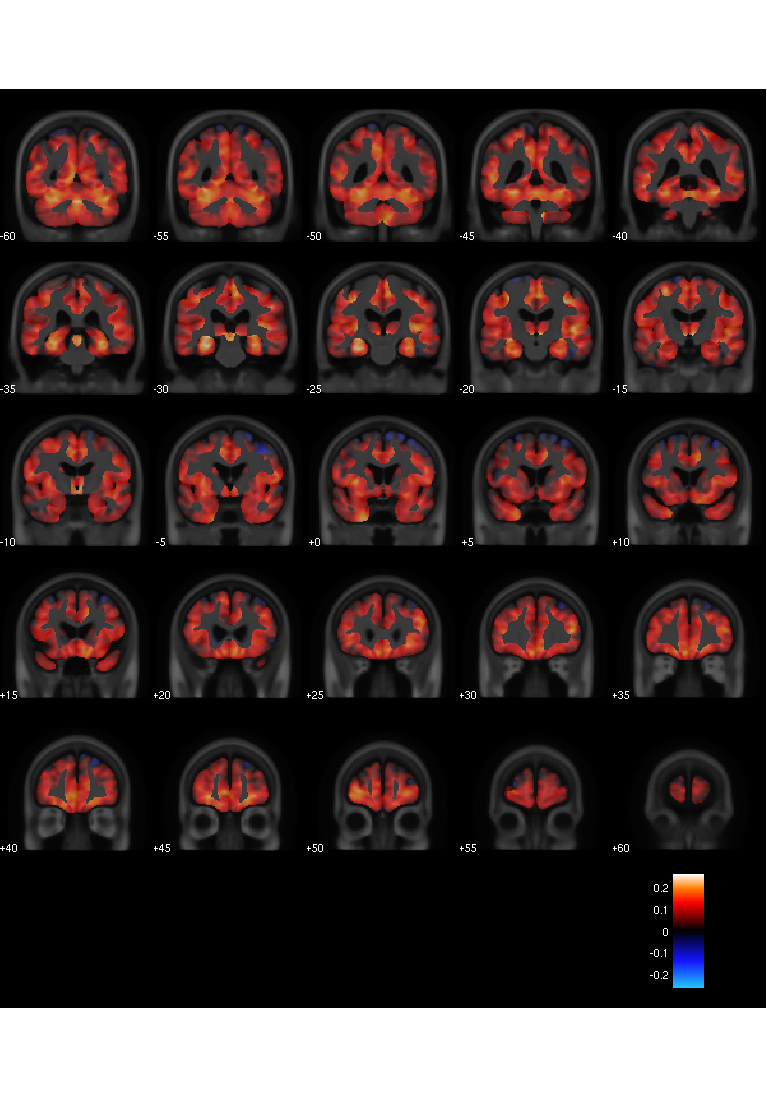

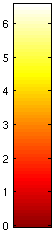

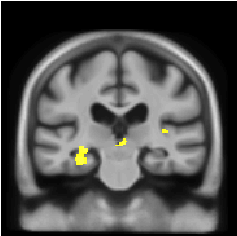

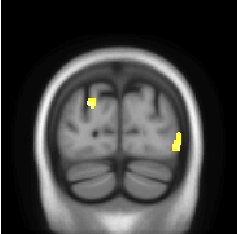

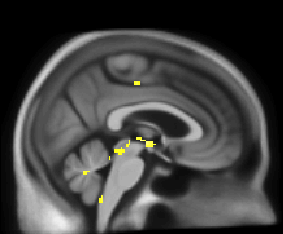

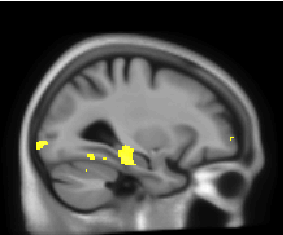

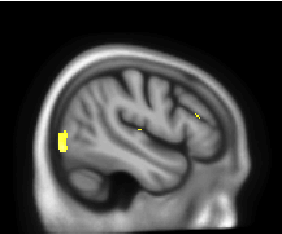

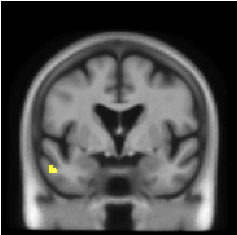

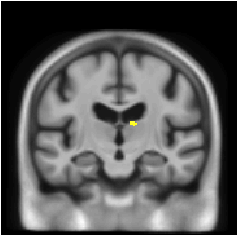

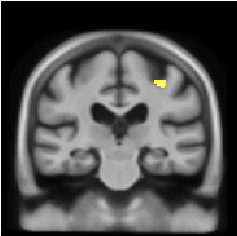

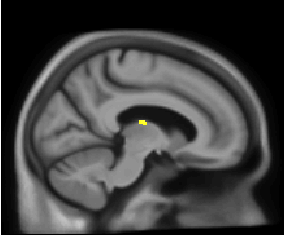

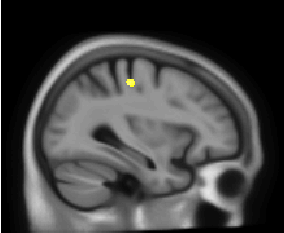

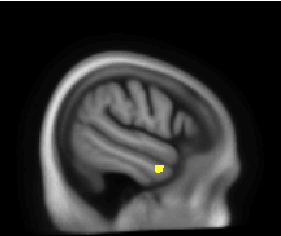

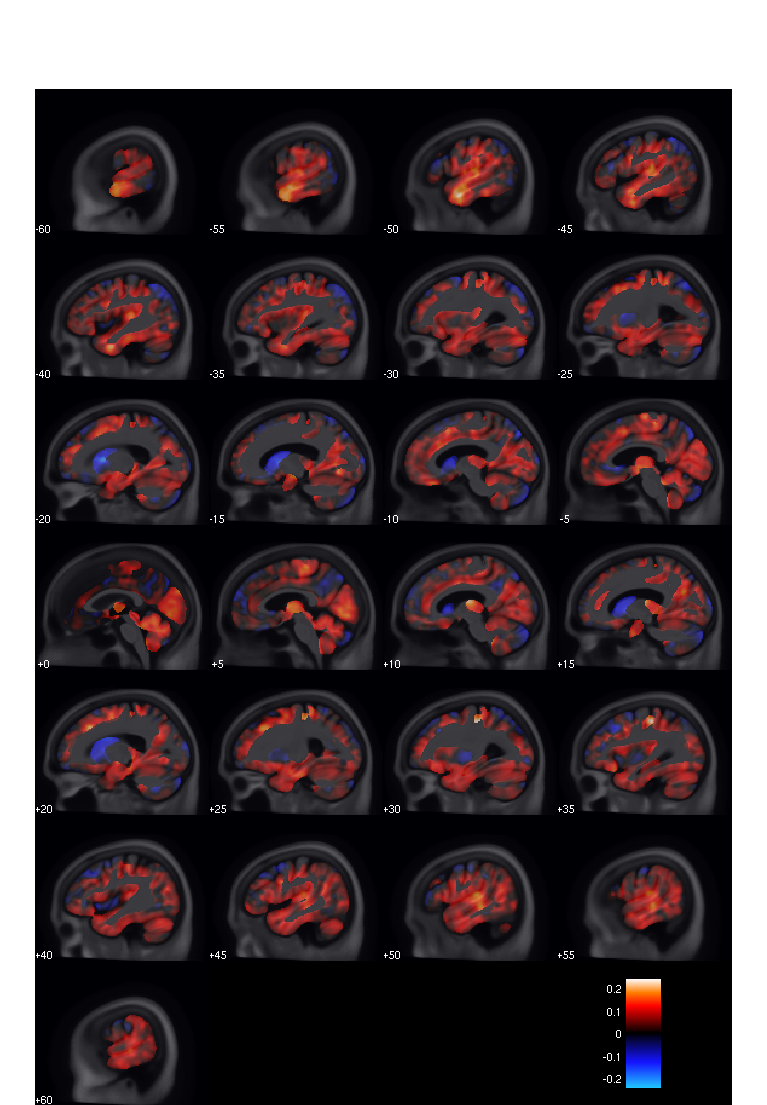

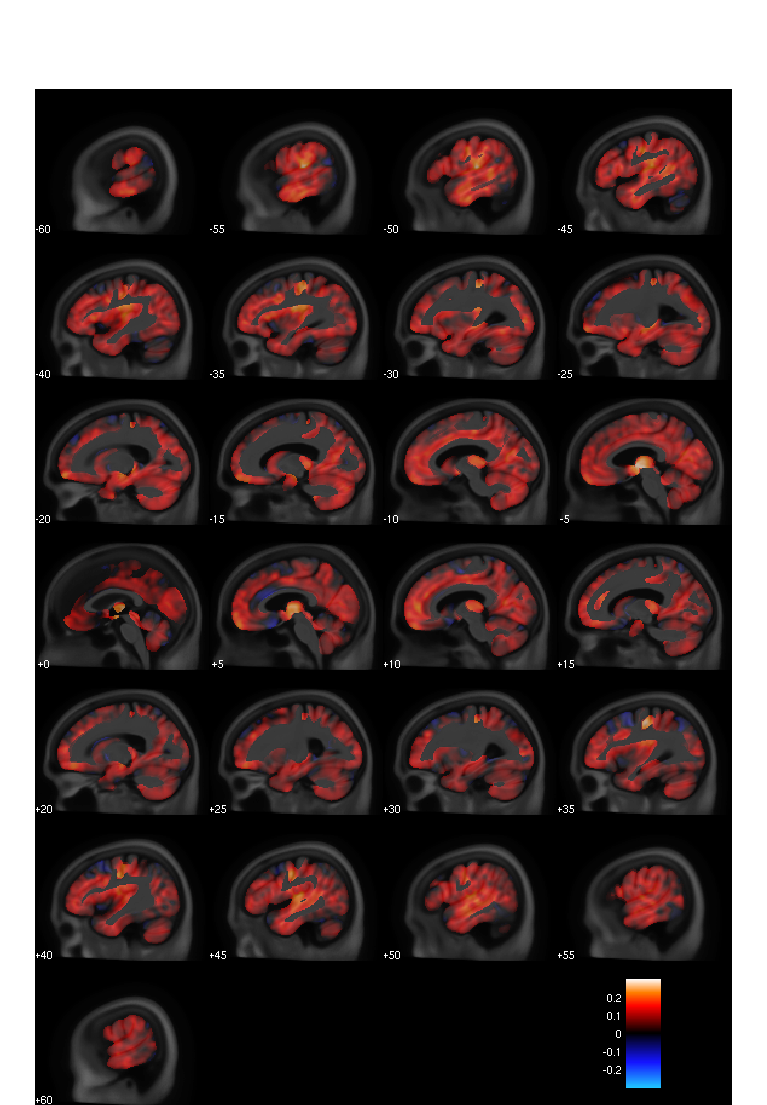

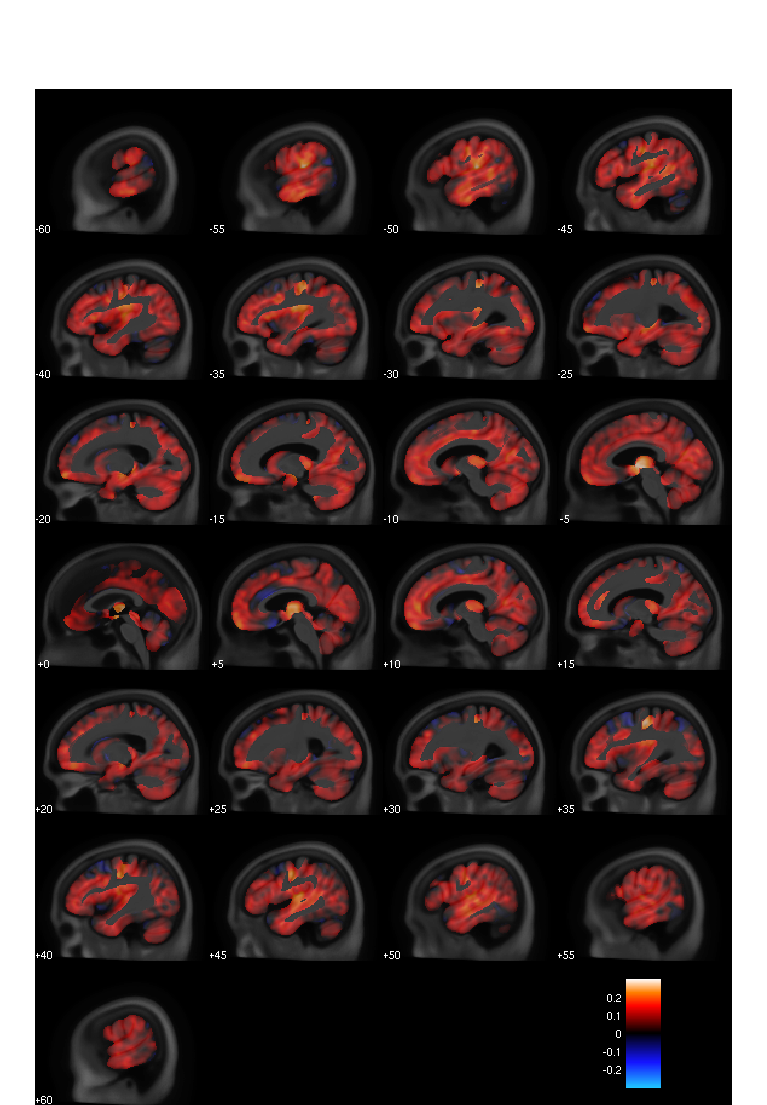

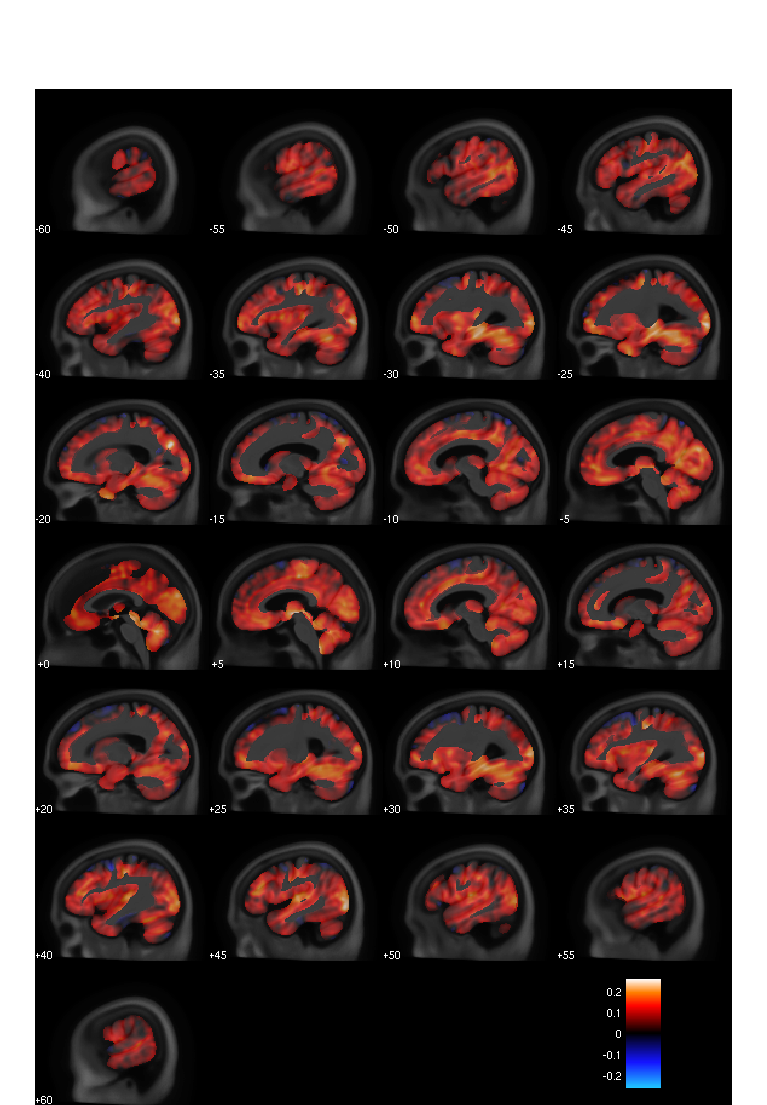

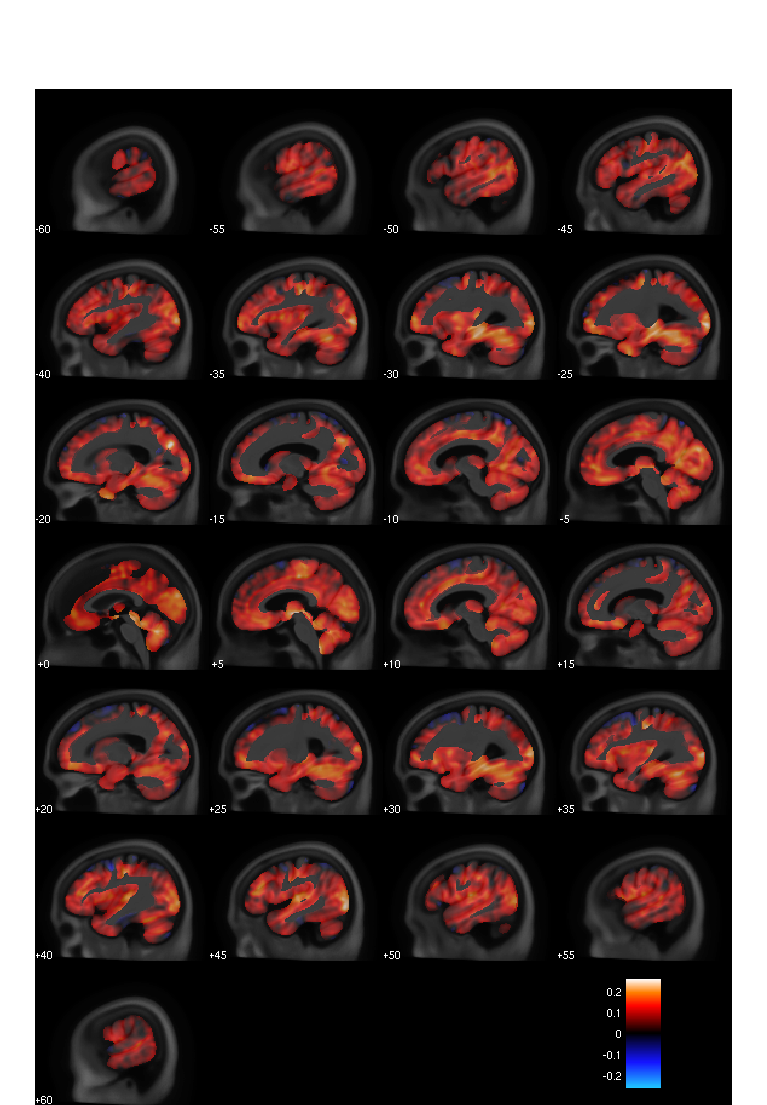

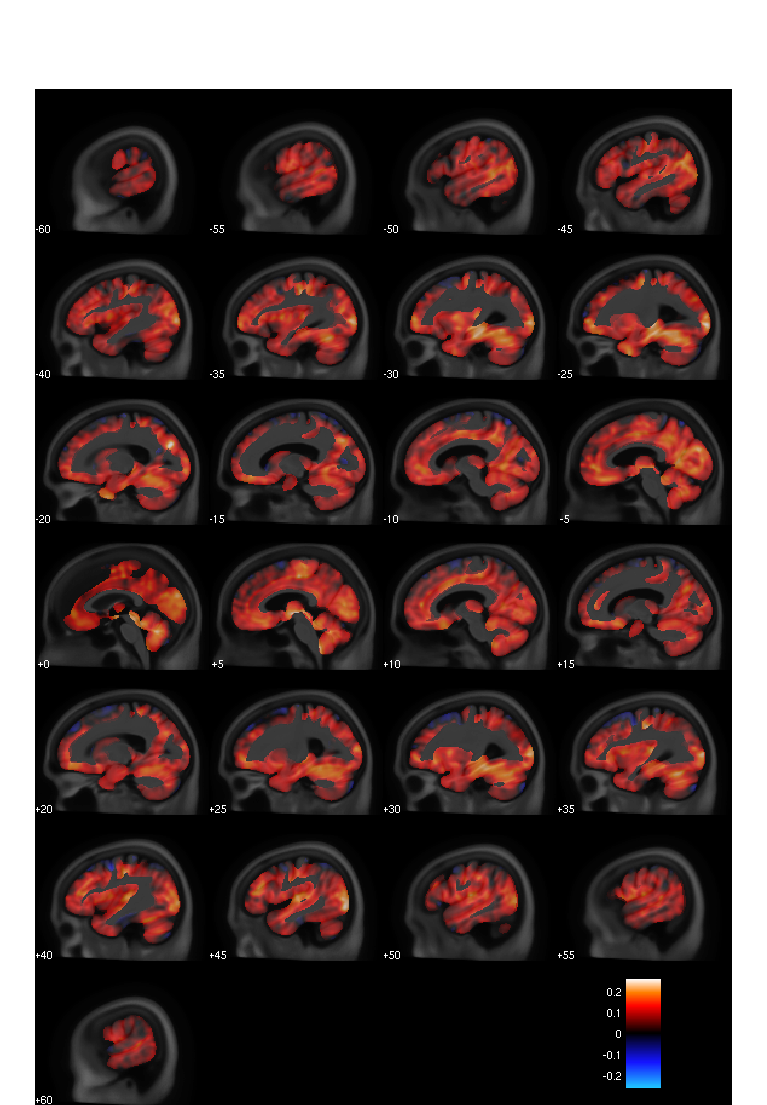

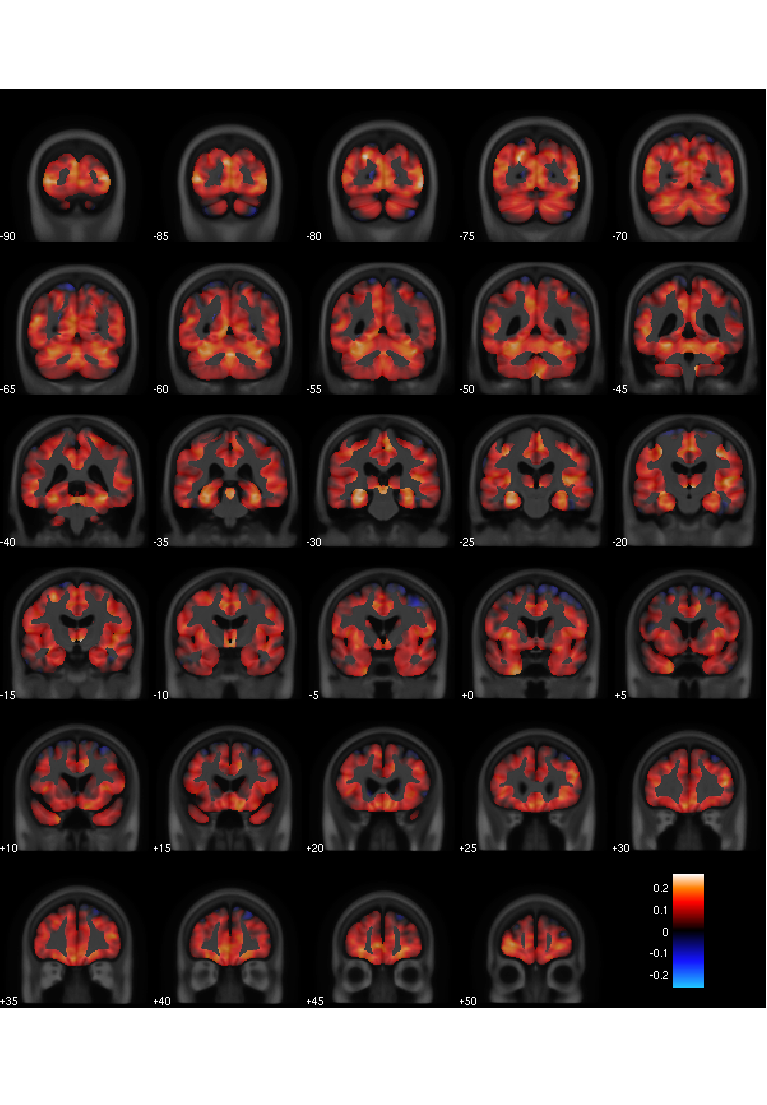

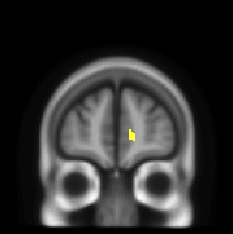

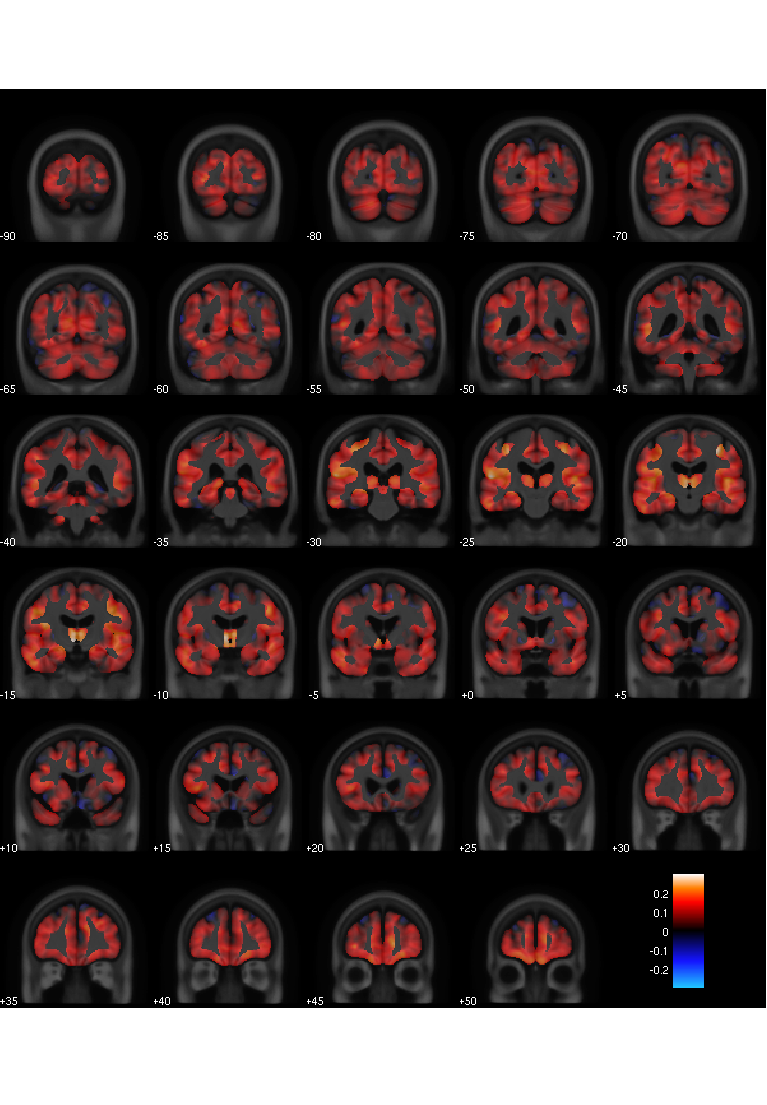

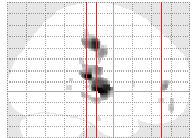

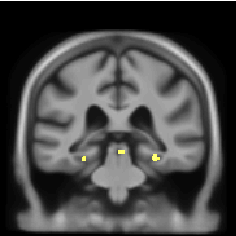

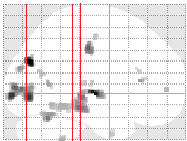

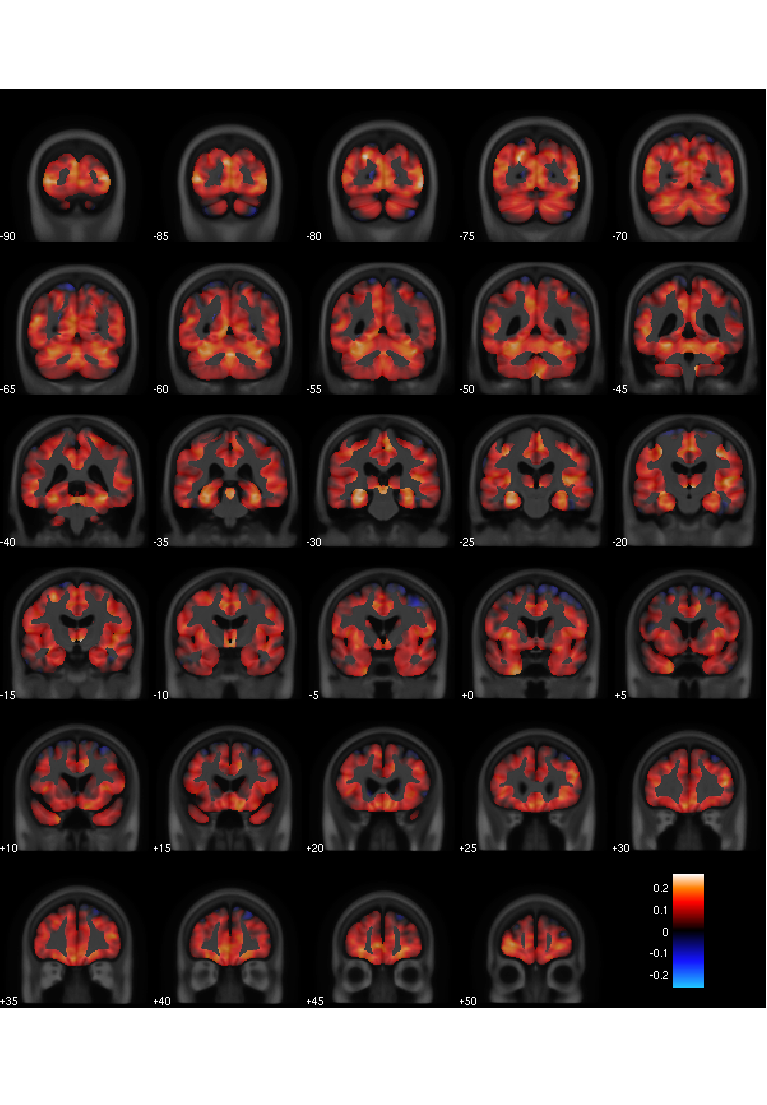


**T Values**

cc values

Supplementary figure 1: Cross-sectional VBM results in the grey matter of control, MCI and AD subjects. Each subject section includes maps of statistical significance (top) showing voxels where baseline log2WMH is associated with lower tissue volume, adjusted for TIV and corrected for multiple comparisons (Family Wise Error (FWE), p<0.05). FWE threshold t=4.59. Effect maps below plot the correlation coefficients (cc map) at each voxel, illustrating the strength of effect across the tissue segments, red indicating WMH positively associated with tissue loss, and blue negative association. Slice positions are depicted on glass brains with corresponding FWE corrected clusters .The 'glass brain' maximum intensity projections should only be used to relate the clusters shown to the slices indicated, not to the glass-brain outline, as the outline is in MNI space while the results are in DARTEL group-average space. Adjacent scales apply to all images.

**Controls**

**Grey Matter**

**MCI**

**Grey Matter**

Grey Matter

**L –––––––––––––––––––––––––––––––––––––––––––––––––––––––––– –––––––––––––––––––––––––––––––––––––––––––––––––––––> R**

**MCI**

**White Matter**

**White Matter**


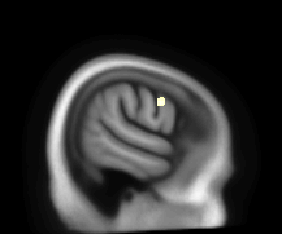

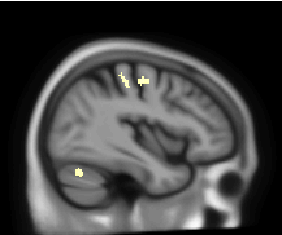

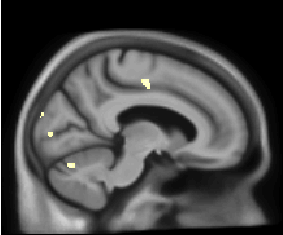

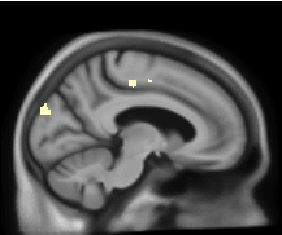

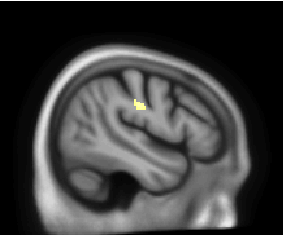

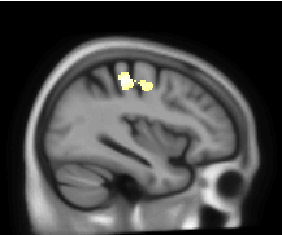

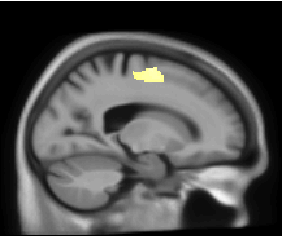

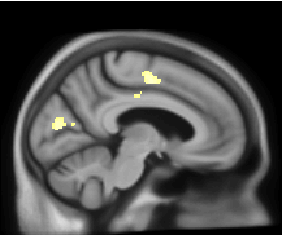

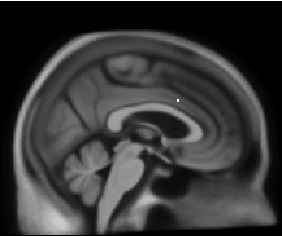

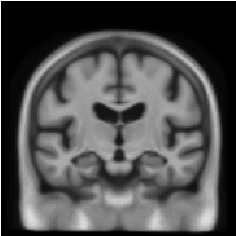

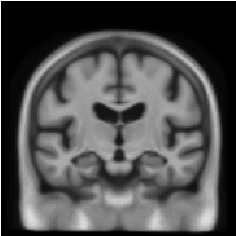

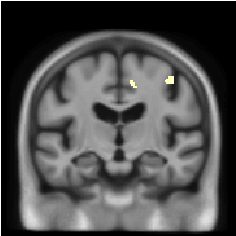

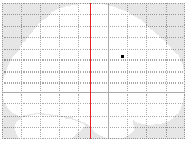

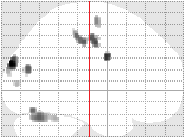

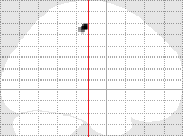

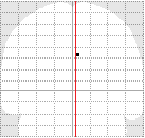

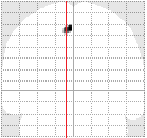

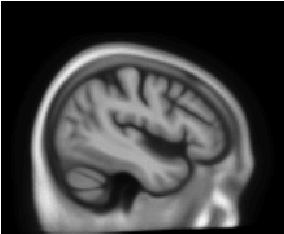

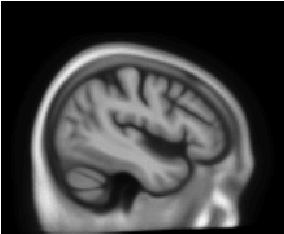

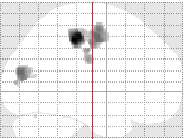

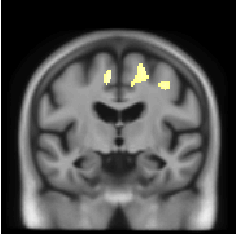

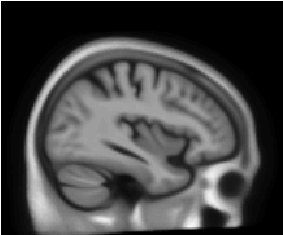

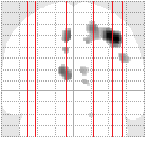

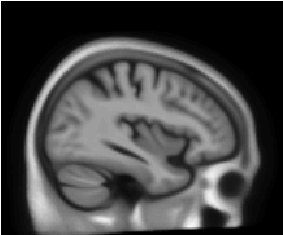

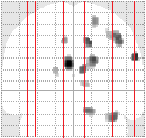

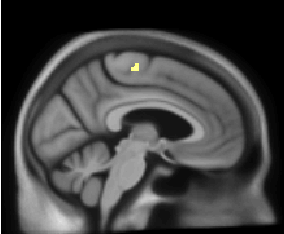

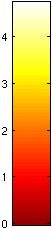

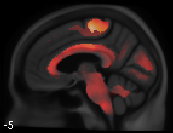

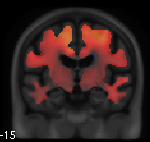

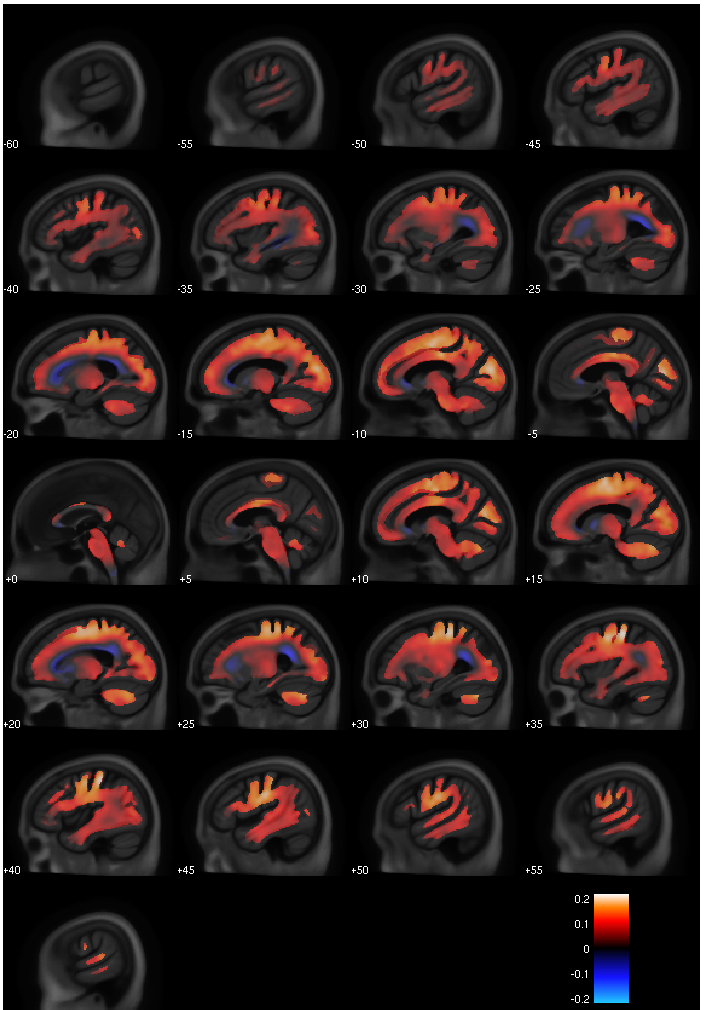

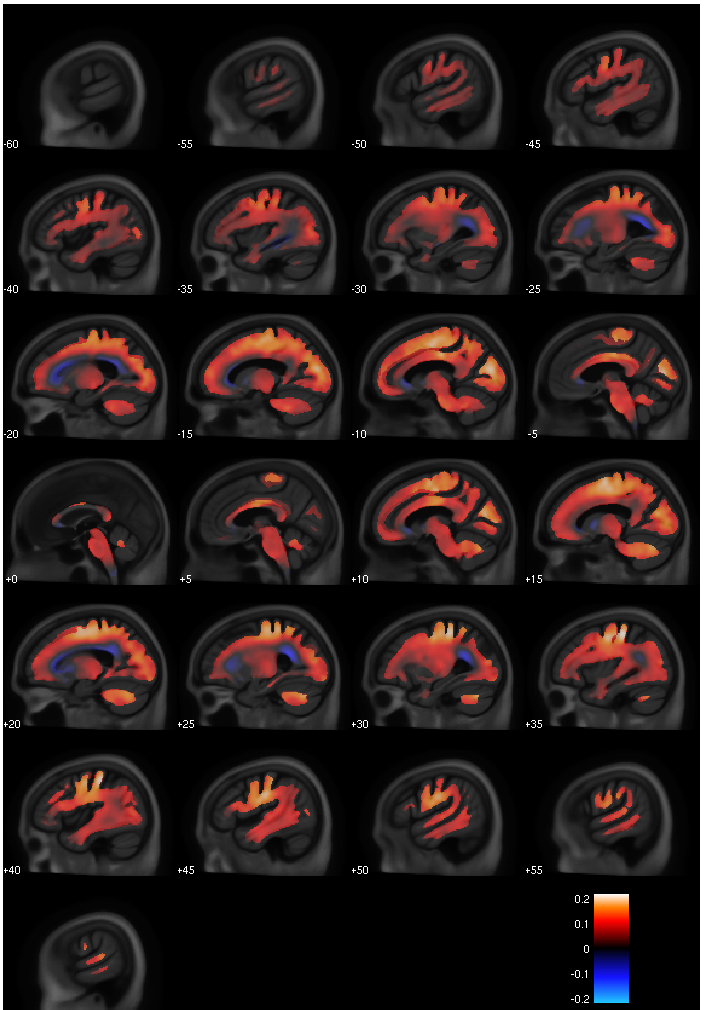

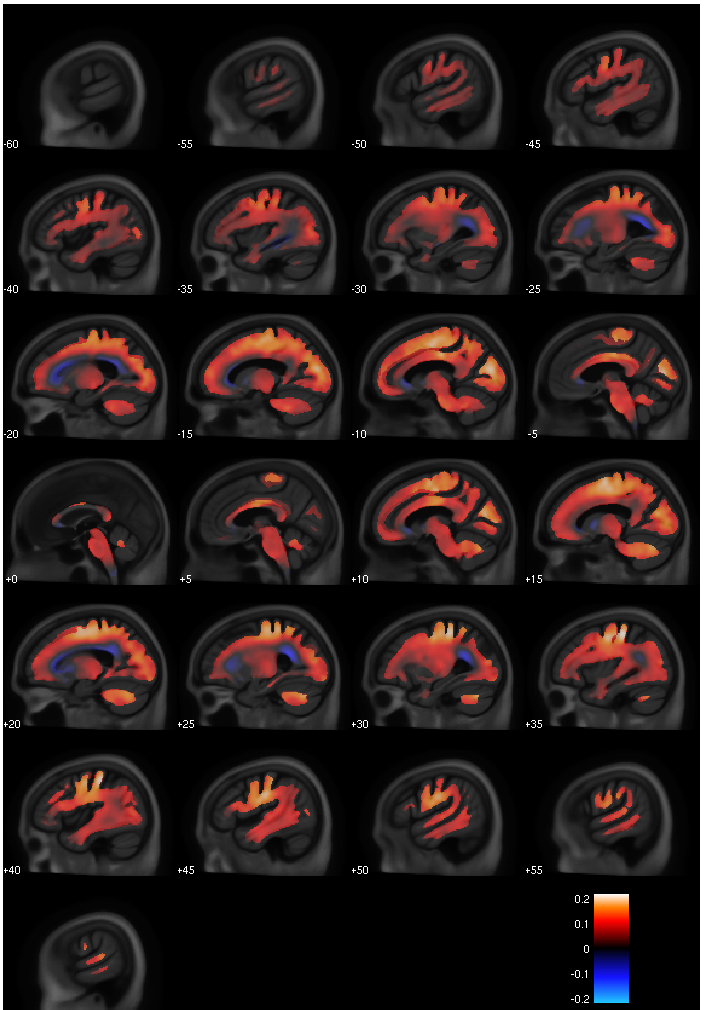

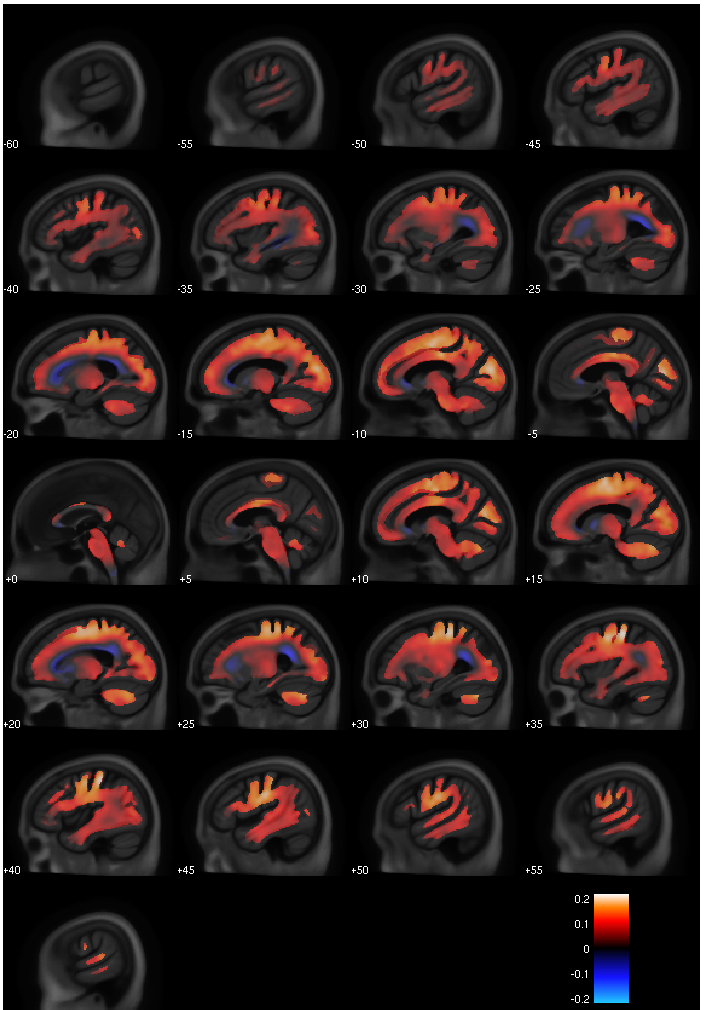

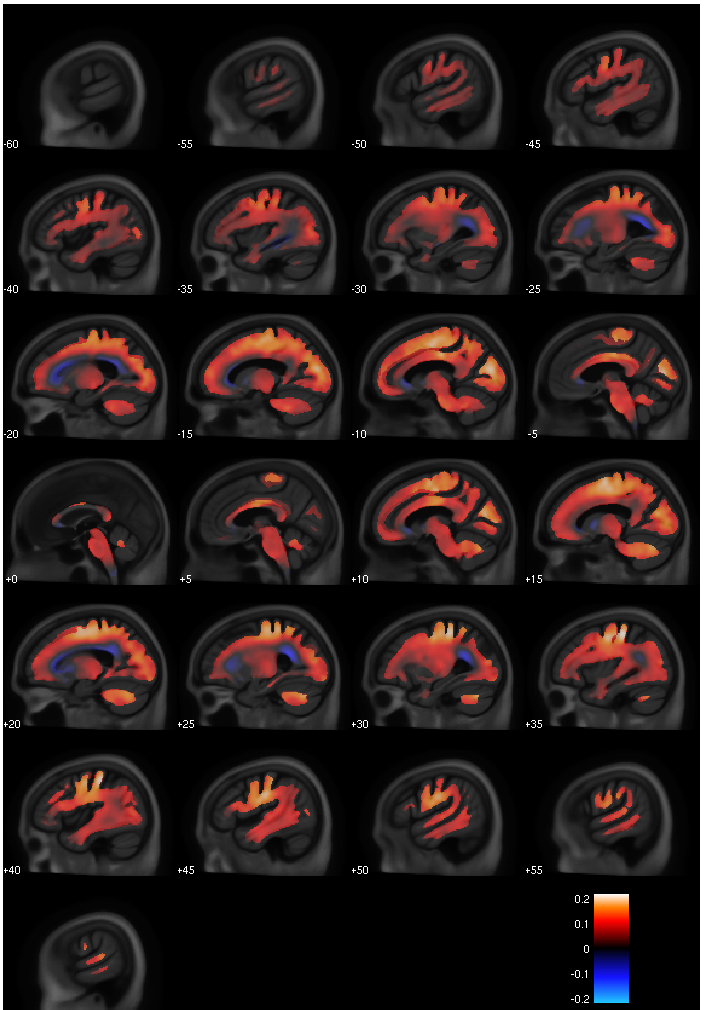

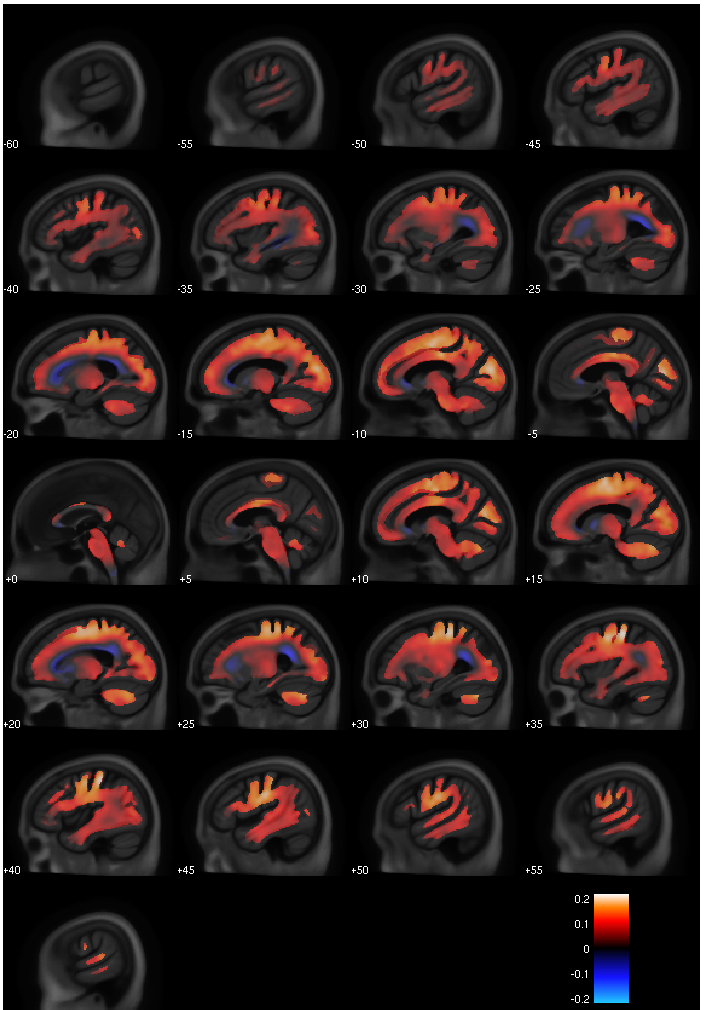

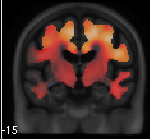

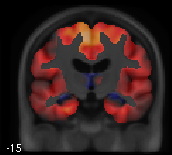

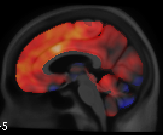

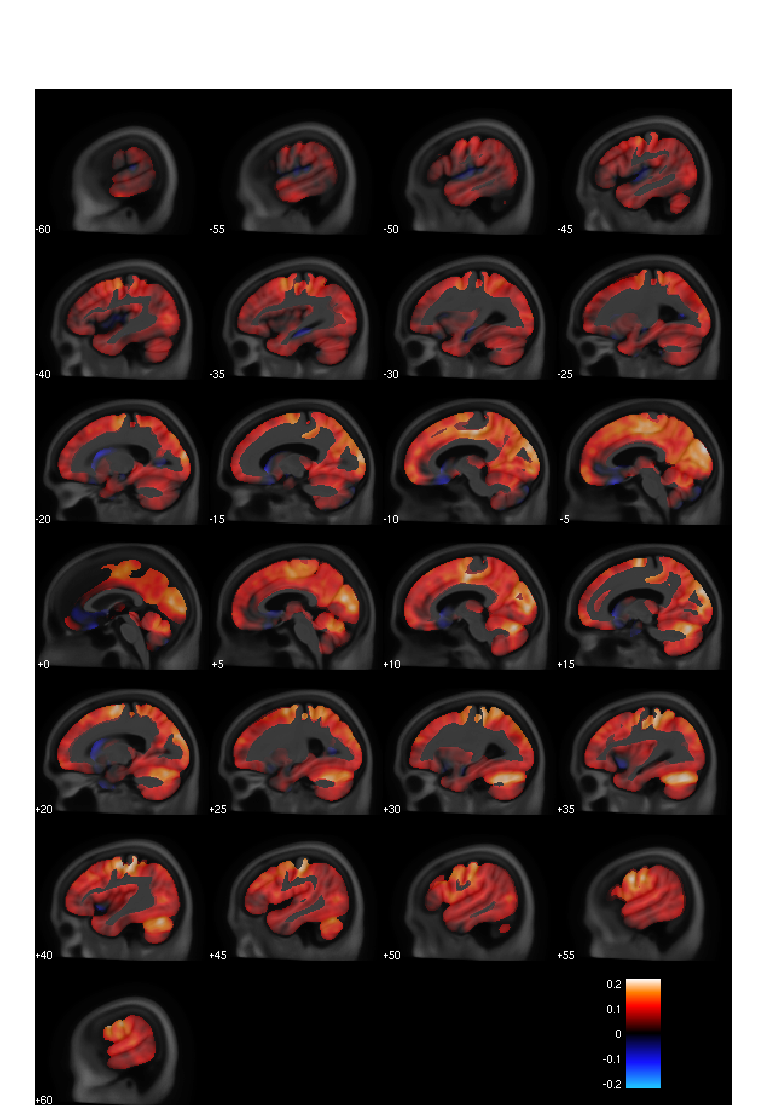

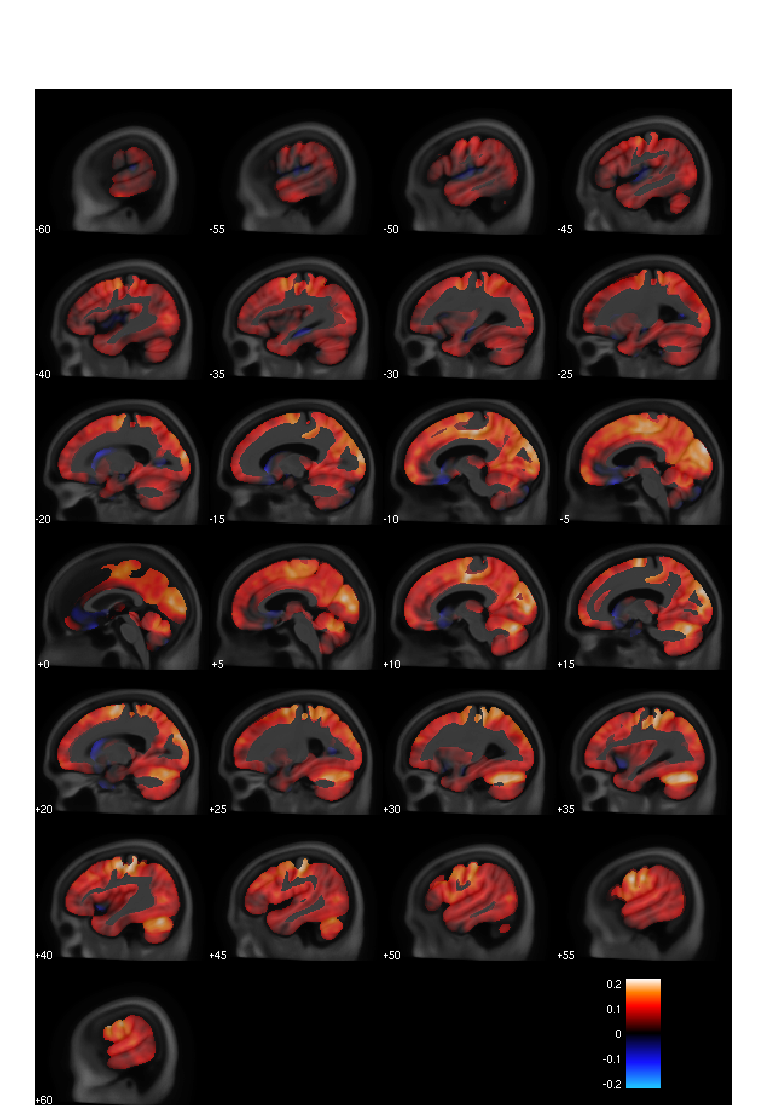

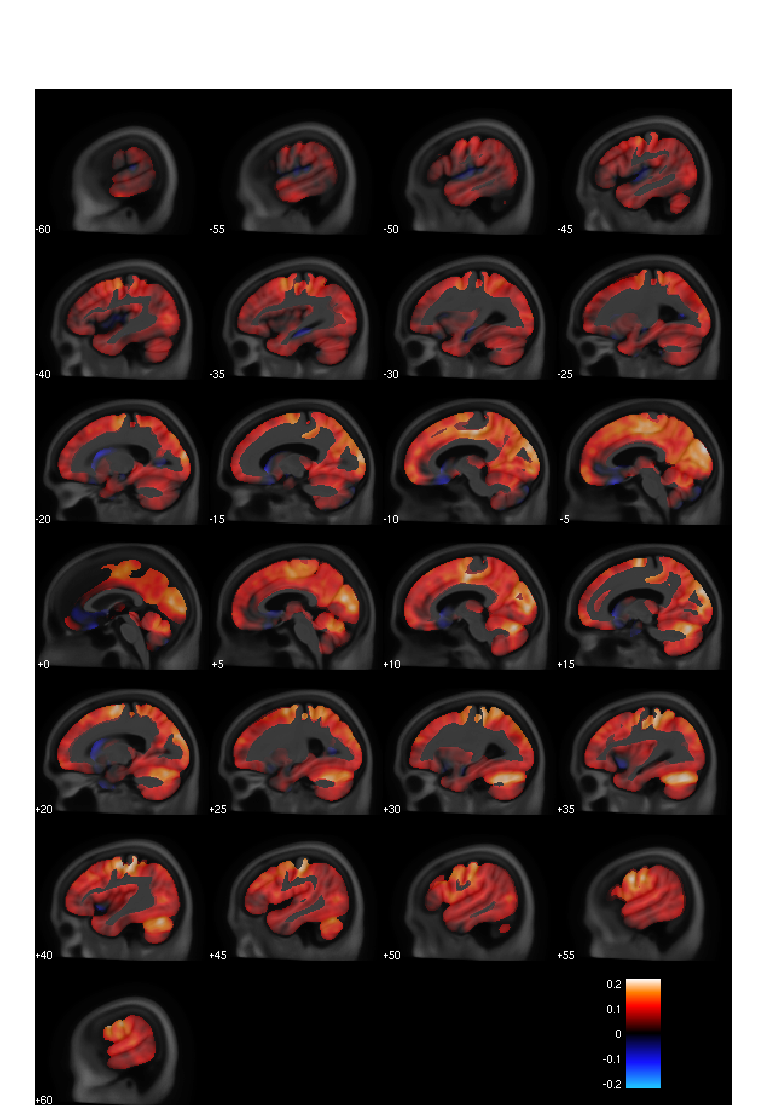

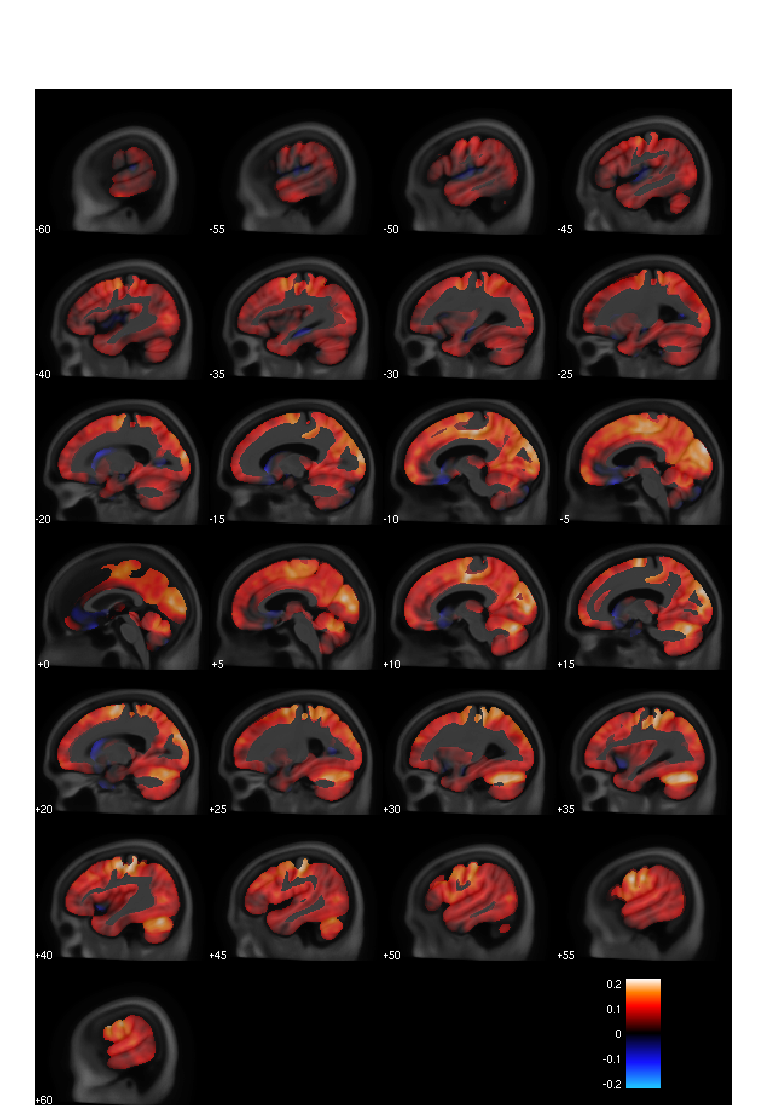

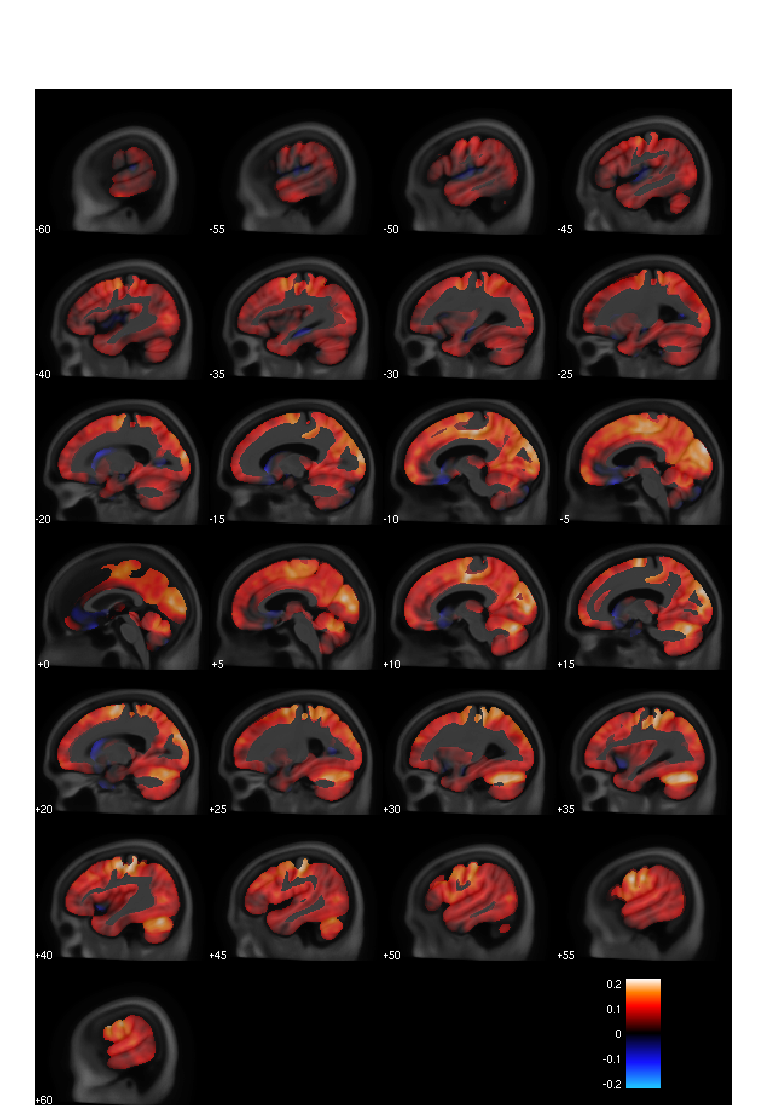

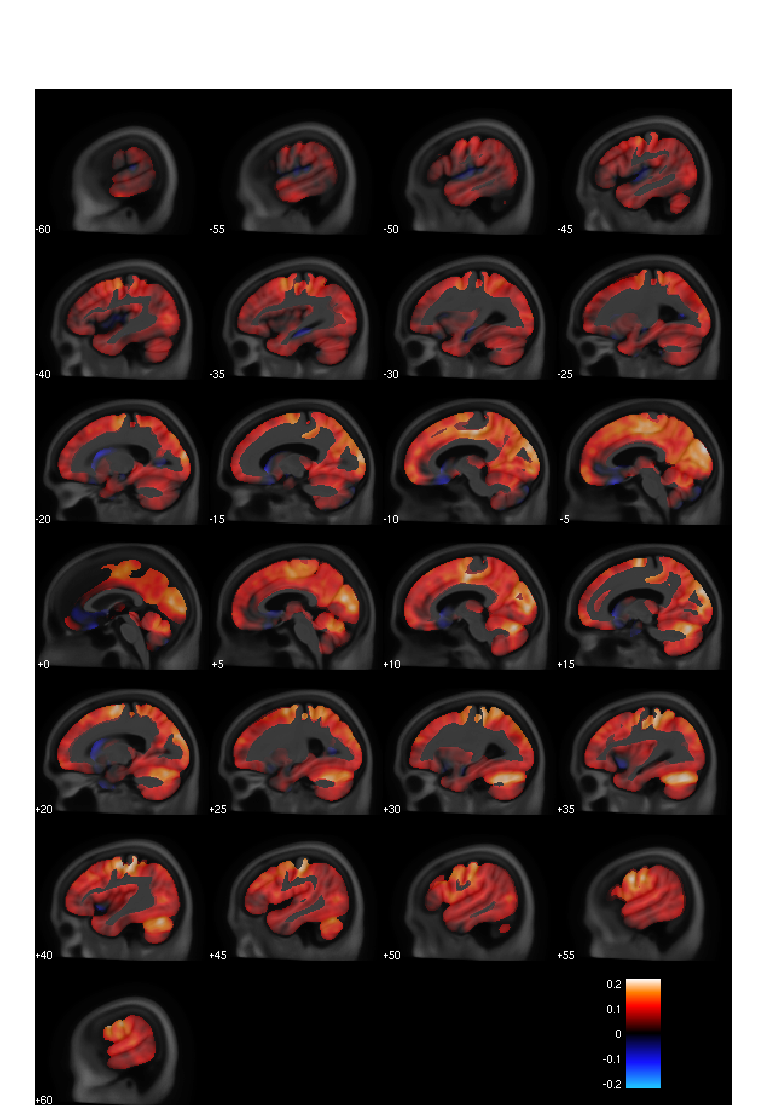

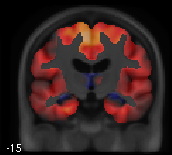

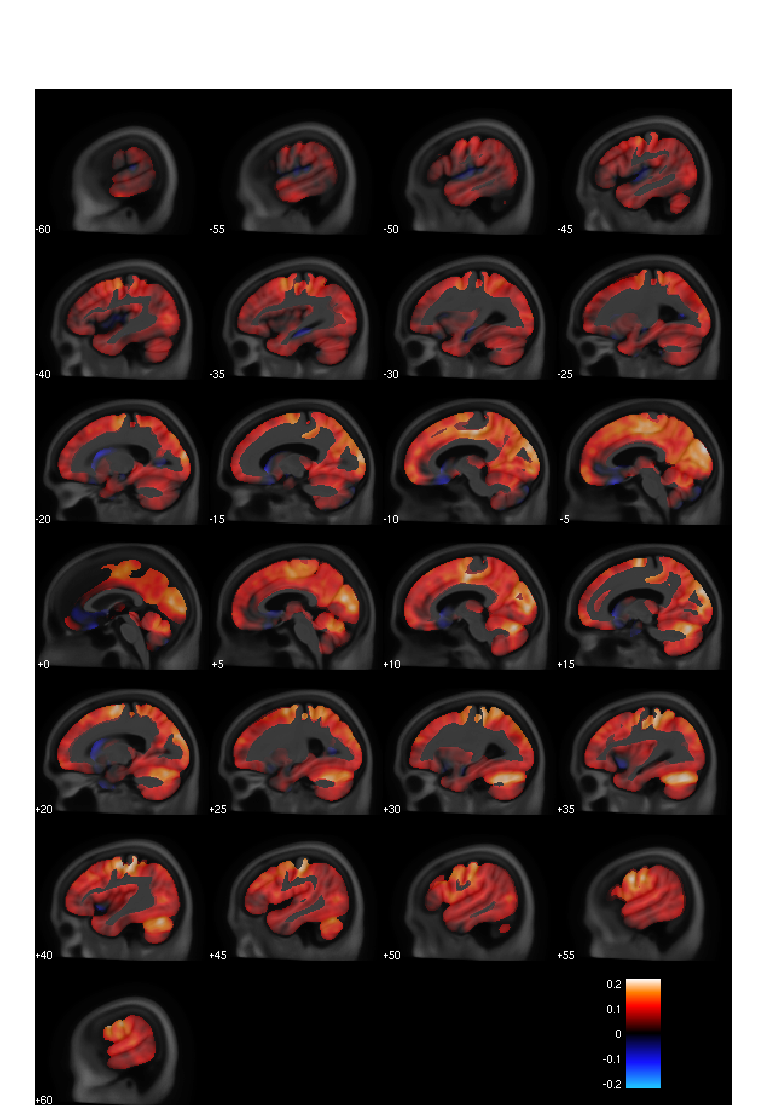


**T Values**

cc values

Supplementary figure 2: Longitudinal VBM results in the grey and white matter of Control and MCI subjects. Each subject section includes maps of statistical significance (top) displaying voxels where baseline log2WMH is associated with decreasing tissue volume, adjusted for TIV and corrected for multiple comparisons (Family Wise Error (FWE), p<0.05). Effect maps below plot the correlation coefficients at each voxel, illustrating the strength of effect, red indicating WMH positively associated with tissue loss, and blue negatively associated. Slice positions are depicted on glass brains with corresponding clusters (also FWE corrected p<0.05). The 'glass brain' maximum intensity projections should only be used to relate the clusters shown to the slices indicated, not to the glass-brain outline, as the outline is in MNI space while the results are in DARTEL group-average space. Scales apply to all images. FWE threshold for grey matter t=4.27, for white matter t=3.99.


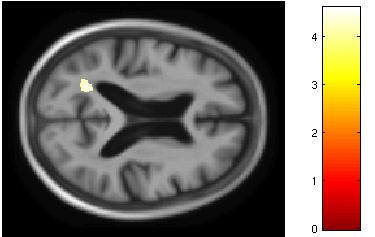

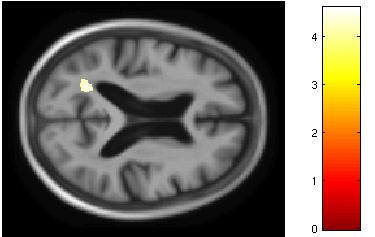


**Alzheimer’s disease**

**Longitudinal**

**White Matter**


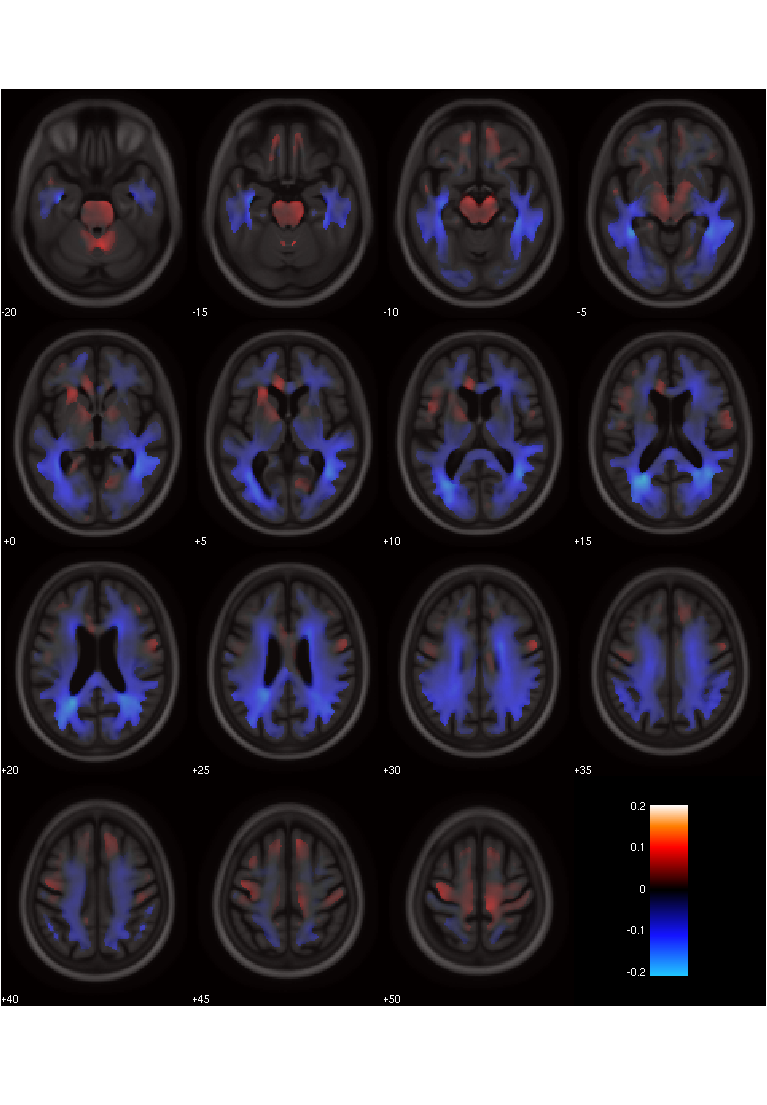

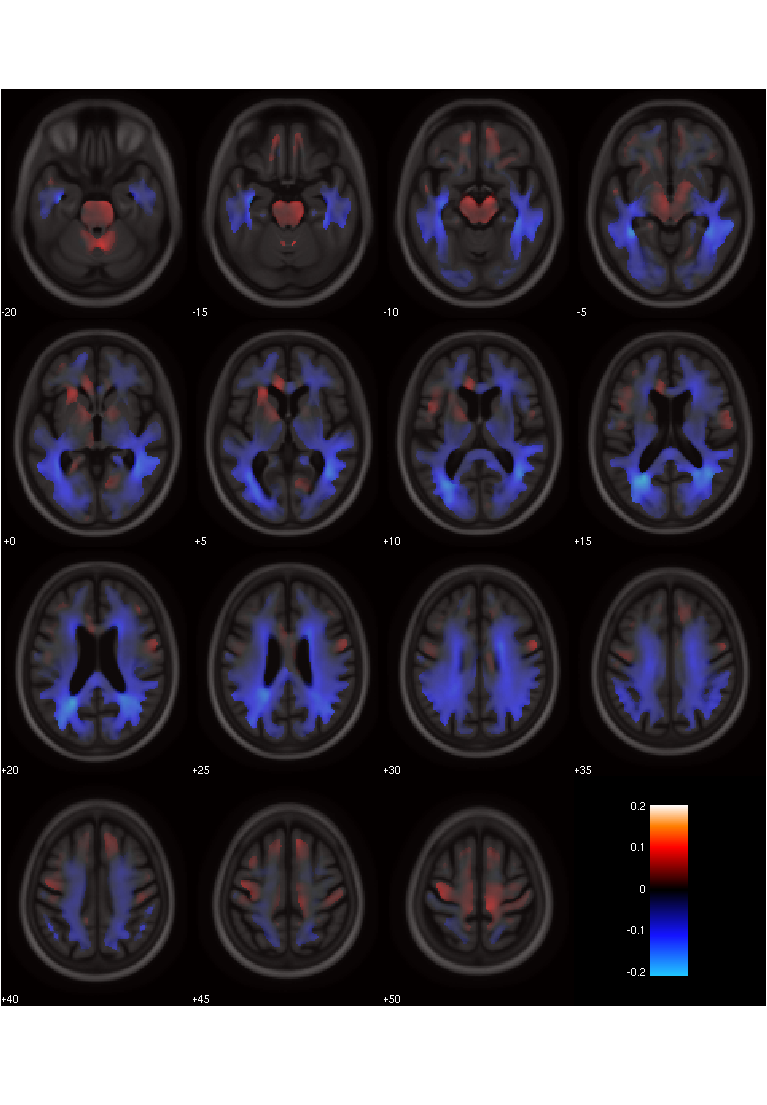

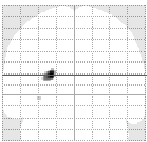


**Controls**

**Cross-Sectional**

**White Matter**


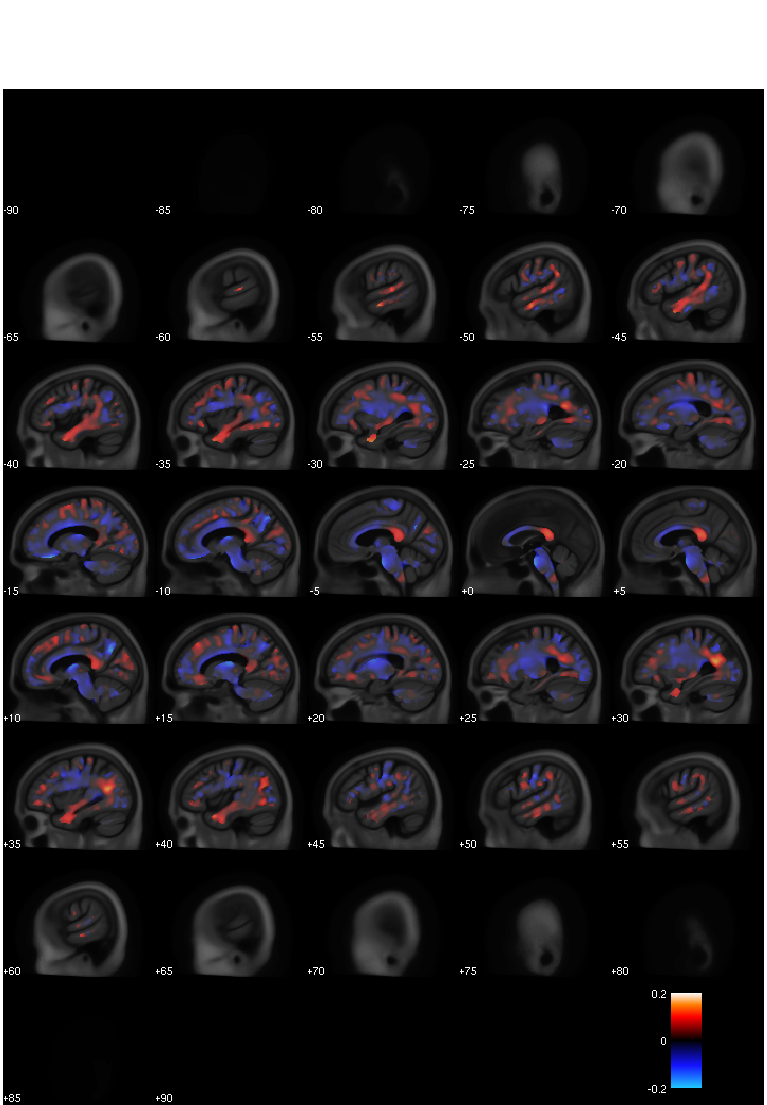

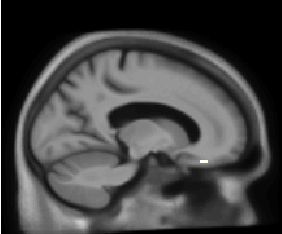

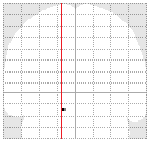


cc values

**T Values**

Supplementary figure 3: Reverse contrast VBM results; where increased log2WMH at baseline is associated with greater cross-sectional volume (top) and more positive (either less atrophy or more expansion) longitudinal volume change (bottom) in controls and Alzheimer’s disease subjects respectively. Effect maps plot the correlation coefficients at each voxel, illustrating the strength of effect, red indicating WMH positively associated with tissue loss, and blue negatively associated. Slice positions are depicted on glass brains with corresponding clusters (also FWE corrected p<0.05). The 'glass brain' maximum intensity projections should only be used to relate the clusters shown to the slices indicated, not to the glass-brain outline, as the outline is in MNI space while the results are in DARTEL group-average space. Scales apply to all images. FWE threshold for cross-sectional t=4.41, for longitudinal t=3.99.

# References

Ashburner, J., Ridgway, G.R., 2012. Symmetric diffeomorphic modeling of longitudinal structural MRI. Front. Neurosci. 6, 197. doi:10.3389/fnins.2012.00197

Ridgway, G.R., Omar, R., Ourselin, S., Hill, D.L.G., Warren, J.D., Fox, N.C., 2009. Issues with threshold masking in voxel-based morphometry of atrophied brains. Neuroimage 44, 99–111. doi:10.1016/j.neuroimage.2008.08.045
